# Quasi-invariance of the Gaussian measure for the two-dimensional stochastic cubic nonlinear wave equation

**DOI:** 10.1007/s40072-025-00407-7

**Published:** 2025-12-09

**Authors:** Justin Forlano, Leonardo Tolomeo

**Affiliations:** 1 School of Mathematics, Monash University, VIC 3800, Australia; 2 School of Mathematics and The Maxwell Institute for the Mathematical Sciences, The University of Edinburgh, James Clerk Maxwell Building, The King’s Buildings, Peter Guthrie Tait Road, EH9 3FD Edinburgh, United Kingdom

**Keywords:** Stochastic nonlinear wave equation, Invariant measure, White noise, Quasi-invariance, Gaussian measure, Absolute continuity, 35L15, 60H15

## Abstract

We consider the stochastic damped nonlinear wave equation $\partial _t^{2}u+\partial _t u+u-\Delta u +u^{3} = \sqrt{2} {\langle {\nabla }\rangle ^{-s}} \xi$ on the two-dimensional torus $\mathbb T^2$, where $\xi$ denotes a space-time white noise and $s>0$. We show that the measure $\vec {\mu }_s$ corresponding to the unique invariant measure for the flow of the associated linear equation is quasi-invariant under the nonlinear stochastic flow.

## Introduction

We consider here the following stochastic damped nonlinear wave equation (SDNLW):

1.1 $$\begin{aligned} {\left\{ \begin{array}{ll} \partial _t^{2}u+\partial _tu+u-\Delta u +u^{3}=\sqrt{2} \langle \nabla \rangle ^{-s} \xi \\ (u,\partial _tu)|_{t = 0} = (u_0,u_1) \end{array}\right. } \qquad ( t, x) \in \mathbb {R}_{+} \times \mathbb {T}^{2}, \end{aligned}$$

where $s>0$, $\xi$ is a space-time white noise, and $\langle \nabla \rangle ^{-s}$ denotes the smoothing operator

1.2 $$\begin{aligned} \langle \nabla \rangle ^{-s}:=(1-\Delta )^{-\frac{s}{2}}. \end{aligned}$$

In the following, we consider (1.1) as a first-order system in the variable ${\textbf{u}}:= (u, v)^{\top }$,

1.3 $$\begin{aligned} {\left\{ \begin{array}{ll} \partial _t {\textbf{u}}= \partial _t\begin{pmatrix} u \\ v \end{pmatrix} = -\begin{pmatrix} 0 &  -1 \\ 1-\Delta &  1 \end{pmatrix} \begin{pmatrix} u \\ v \end{pmatrix} - \begin{pmatrix} {0}\\ {u^3} \end{pmatrix} +\begin{pmatrix} {0}\\ {\sqrt{2} \langle \nabla \rangle ^{-s}\xi },\end{pmatrix} \\ {\textbf{u}}|_{t = 0} = {\textbf{u}}_0 = \begin{pmatrix} u_0 \\ u_1 \end{pmatrix}. \end{array}\right. } \end{aligned}$$

The interest for the equation stemmed from recent results pertaining to the PDE construction of the Euclidean $\Phi ^4$ quantum field theory [1, 2, 12, 24, 25, 34]. Namely, if one considers the stochastic quantisation equation

1.4 $$\begin{aligned} \partial _t u + u - \Delta u = -u^3 + \sqrt{2}\xi , \end{aligned}$$

as time goes to infinity, we expect a generic solution to (1.4) to converge to the (massive) $\Phi ^4$-measure.

In the above context, the stochastic wave equation

1.5 $$\begin{aligned} \partial _t^2 u + \partial _t u + u - \Delta u = - u^3 + \sqrt{2} \xi , \end{aligned}$$

corresponds to the so-called canonical stochastic quantisation equation, or equivalently, to the kinetic Langevin equation for the $\Phi ^4$-measure with momentum $v = \partial _t u$. Therefore, one can sample the $\Phi ^4$-measure by sampling the law of the first component of the solution of (1.5). This procedure has the name of Hamiltonian Montecarlo (HCM). It has numerically been observed that in the finite dimensional setting, HCM converges faster than MCMC (Markov Chain Montecarlo) in many situations, see [15] (and references within), for a rigorous justification of these faster convergence rates.

As a consequence, equation (1.5) has received a substantial amount of attention in recent years, as an alternative approach to stochastic quantisation of the $\Phi ^4$ measure. However, due to the worse analytical properties of the wave propagator $(\partial _t^2 - \Delta )^{-1}$ compared to those of the heat propagator $(\partial _t - \Delta )^{-1}$, there are far fewer results available, namely, uniqueness and convergence to the invariant measure have been proven by the second author only in [52] for dimension $d = 1$ and in [53] for dimension $d = 2$. Local and global well posedness results for related models, but without uniqueness of the measure, can be found in [26, 27, 36, 37, 43, 51].

In light of the above discussion, we see the equation (1.1) as a version of (1.5) in fractional dimension $d = 2-2 s$. A similar approach to fractional dimension has already been considered for the parabolic case of (1.4), see for instance [9]. In the work by the authors [19], it has been shown that equation (1.1) also has a unique invariant measure, to which we have convergence for every initial data belonging to a certain class. However, strictly speaking, the equation (1.1) is not a Langevin equation, because of the presence of the smoothing operator $\langle \nabla \rangle ^{-s}$. This prevents us from writing an explicit formula for the invariant measure, which in turn leaves many open questions. A fairly natural question to ask is the following. Denoting by $\vec {\mu }_s$ the invariant measure for the linear equation

$$\begin{aligned} \partial _t^{2}u+\partial _tu+u-\Delta u =\sqrt{2} \langle \nabla \rangle ^{-s} \xi , \end{aligned}$$

or more precisely, to the system

$$\begin{aligned} {\left\{ \begin{array}{ll} \partial _t {\textbf{u}}= \partial _t \begin{pmatrix} u \\ v \end{pmatrix} = -\begin{pmatrix} 0 &  -1 \\ 1-\Delta &  1 \end{pmatrix} \begin{pmatrix} u \\ v \end{pmatrix} +\begin{pmatrix} {0}\\ {\sqrt{2} \langle \nabla \rangle ^{-s}\xi }, \end{pmatrix} \\ {\textbf{u}}|_{t = 0} = {\textbf{u}}_0 = \begin{pmatrix} u_0 \\ u_1 \end{pmatrix}. \end{array}\right. } \end{aligned}$$

is it true that the invariant measure $\vec {\rho }_s$ of (1.1) satisfies $\vec {\rho }_s \ll \vec {\mu }_s$? As opposed to the invariant measure for the nonlinear equation, the measure $\vec {\mu }_s$ has an explicit formula, namely, it formally holds that

1.6 $$\begin{aligned} \vec {\mu }_s = Z^{-1} \exp \Big (- \frac{1}{2} \Vert u\Vert _{H^{1+s}}^2 - \frac{1}{2} \Vert v\Vert _{H^s}^2\Big )du\,dv. \end{aligned}$$

Therefore, this absolute continuity property would allow us to bootstrap relevant properties of the invariant measure from the analogous properties of $\vec {\mu }_s$. However, while the analogous of this statement is relatively simple to show for the parabolic equation (1.4), for the wave case, it turns out to be a very difficult problem. Indeed, even in the case of the stochastic Navier-Stokes equation on $\mathbb {T}^2$,

1.7 $$\begin{aligned} \partial _t u = \Delta u - u \cdot \nabla u + \langle \nabla \rangle ^{-s} \xi , \end{aligned}$$

despite the fact that the existence of the invariant measure has been proven in the ’90s in [16, 17], this question has been open until very recently, when Coe, Hairer and the second author gave a positive answer in [11].^1^

A closely related question is the following: if ${{\,\textrm{Law}\,}}({\textbf{u}}_0) = \vec {\mu }_s,$ is it true that ${{\,\textrm{Law}\,}}({\textbf{u}}(t)) \ll \vec {\mu }_s$? If this happens, we say that the measure $\vec {\mu }_s$ is *quasi-invariant* under the (stochastic) flow of (1.3). In the parabolic case of (1.4) and (1.7), or more in general, whenever one has the strong Feller property, the two questions are actually equivalent. Unfortunately, the strong Feller property fails for equation (1.3), and one cannot deduce absolute continuity of the invariant measure from quasi-invariance.

Quasi-invariance for the deterministic flow of Hamiltonian and dispersive PDEs in situations where the initial data is not distributed according to an invariant measure has been studied extensively in the last decade. This pursuit was initiated by Tzvetkov in [54] for the BBM equation, with many results following for a number of other Hamiltonian PDEs, see [7, 10, 14, 18, 20–23, 29, 32, 38–42, 44–46, 49, 54] and references therein for a list of results in this direction. We point out the results in [29, 42, 46] prove quasi-invariance results in the case of the deterministic and undamped version of (1.1).

Nevertheless, the question of quasi-invariance for stochastic dispersive models is yet to be answered. In this paper, we prove the first of such results; in particular, we show the following.

### Theorem 1.1

For each $s>0$, let $\Phi _{t}({\textbf{u}}_0;\xi )$ denote the solution to SDNLW (1.1) as in (3.2), and suppose that ${{\,\textrm{Law}\,}}({\textbf{u}}_0 )= \vec {\mu }_{s}$. Then for each $t\ge 0$, the probability measure $Law (\Phi _{t}({\textbf{u}}_0;\xi ))$ is absolutely continuous with respect to $\vec {\mu }_{s}$.

While, as mentioned, this result is not strong enough to show that $\vec {\rho }_s \ll \vec {\mu }_s$, this result immediately implies the following structural statement about the invariant measure for (1.1).

### Theorem 1.2

Let $\rho _s$ be the invariant measure for (1.1). Then either we have $\vec {\rho }_s \ll \vec {\mu }_s$, or $\vec {\rho }_s \perp \vec {\mu }_s$.

Therefore, in order to show that $\vec {\rho }_s \ll \vec {\mu }_s$, one just needs to exclude the situation in which $\vec {\rho }_s \perp \vec {\mu }_s$. If one has the strong Feller property, this is immediately true due to support theorems, since $\vec {\mu }_s$ has full support (for any reasonable topology on the space of initial data). However, also due to the failure of the strong Feller property for (1.1), in this case it is very non-trivial to exclude the situation in which $\vec {\rho }_s$ and $\vec {\mu }_s$ are mutually singular.

### Outline of the proof

The proof of Theorem 1.1 will follow closely the strategy of the deterministic results in [29, 42, 46], hence we first describe how one obtains quasi-invariance of the measure $\vec {\mu }_s$ for the deterministic equation

1.8 $$\begin{aligned} \partial _t^2 u + (1 - \Delta ) u + u^3 = 0. \end{aligned}$$

One can formally see this equation as an infinite-dimensional system of ODEs in the form

$$\begin{aligned} \dot{y} = b(y), \end{aligned}$$

with $\text {div}(b) = 0$ on some infinite-dimensional vector space *X*. Therefore, denoting by $\Phi _t$ the flow of this system of ODEs, for a measure $\nu _0 = \exp \big ( - E(x)\big )dx$ and for any Borel set *A*, one has that

$$\begin{aligned} \frac{d}{dt} \nu _0(\Phi _t(A)) = \int _{\Phi _t(A)} - \frac{d}{dt}\left. E(\Phi _t(x))\right| _{t=0} d\nu _0(x). \end{aligned}$$

This allows to perform a Gronwall-type argument for the quantity $\nu _0(\Phi _t(E))$, from which one obtains that quasi-invariance holds whenever for every ball $B_R \subset X$, there exists some $\eta _R > 0$ such that

1.9 $$\begin{aligned} \int _{B_X(0, R)} \exp \Big (\eta _R \big |\frac{d}{dt}\left. E(\Phi _t(x))\right| _{t=0}\big |\Big ) d \nu _0 < \infty . \end{aligned}$$

While not codified in this way, one can find a proof of this result in [44]. As the condition (1.9) does not hold for the measures $\vec {\mu }_s$, what is shown in [29, 42, 46] is that, for an appropriate range of $s>0$, if one defines the energy functional

$$\begin{aligned} E(u,v) = \frac{1}{2} \Vert u\Vert _{H^{1+s}}^2 - \frac{1}{2} \Vert v\Vert _{H^s}^2 - \frac{3}{2} \int \big ((\langle \nabla \rangle ^s u)^2 - \sigma \big ) u^2 \end{aligned}$$

with $$\sigma = \mathbb {E}_{\vec {\mu }_s} \Vert \langle \nabla \rangle ^s u\Vert _{L^2}^2$$, then

$$\begin{aligned} \vec {\mu }_s \ll Z^{-1} \exp (- E(u,v)) dudv \ll \vec {\mu }_s , \end{aligned}$$

and the property (1.9) holds.^2^

In order to prove Theorem 1.1, we follow a similar approach in the stochastic setting. We define the measure

$$\begin{aligned} \vec {\nu }_s \sim \exp \Big ( - \frac{1}{2} \Vert u\Vert _{H^{1+s}}^2 - \frac{1}{2} \Vert v\Vert _{H^s}^2 - \frac{3}{2} \int \big ((\langle \nabla \rangle ^s u)^2 - \sigma \big ) u^2 \Big )dudv, \end{aligned}$$

and we try to show that under a condition analogous to (1.9), we can deduce quasi-invariance of the measure $\vec {\nu }_s$ in the sense of Theorem 1.1. Indeed, in Section 5, we first prove that the measure $\vec {\nu }_s$ satisfies the alternative condition

1.10 $$\begin{aligned} \int _{B_X(0, R)} \exp \Big (\eta _R \big |\frac{d}{dt}\left. \mathbb {E}_\xi \big [E(\Phi _t({\textbf{u}}_0, \xi ))\big ]\right| _{t=0}\big |\Big ) d \nu _0({\textbf{u}}_0) < \infty , \end{aligned}$$

and then we prove that this condition can indeed replace (1.9) in the stochastic setting, thus completing the proof. While the argument is specialised to equation (1.1), we believe that a similar condition can be proven more in general.

### Further remarks

#### Remark 1.3

In the proof of the condition (1.10), we follow a slightly different approach from the cited works [42, 46]. This allows us to prove that the estimate holds in the full non-singular regime $s >0$. When applied to the deterministic wave equation (1.8), this allows us to show the estimate (1.9), and thus quasi-invariance of $\vec {\mu }_s$, in the regime $s>0$, thus improving the result in [46], which instead requires $s > \frac{5}{2}$.

#### Remark 1.4

One might wonder if a result analogous to Theorem 1.1 holds for the *focusing* equation

$$\begin{aligned} \partial _t^{2}u+\partial _tu+u-\Delta u -u^{3}=\sqrt{2} \langle \nabla \rangle ^{-s} \xi , \end{aligned}$$

where we flipped the sign of the nonlinearity. In principle, the arguments of this paper apply mutatis mutandis to this equation as well, with one crucial difference: due to the different sign of the nonlinearity, there is no available global well-posedness theory for this equation, and in fact, we expect solutions to blow up in finite time with probability 1 for every initial data. Therefore, we expect the analogous of Theorem 1.1 to hold only after conditioning on the event where the flow has not blown up before time *t*. Moreover, due to the sign of the nonlinearity, the construction of the measure $\vec {\nu }_s$ in Section 4 is way more delicate in this case. In particular, there is no way of making $\vec {\nu }_s$ into a probability measure, so one has to work with $\sigma$-finite measures throughout the proof, with all the related technical complications.

#### Remark 1.5

In the deterministic case (1.8), it is in principle possible to write an explicit formula for the Radon-Nikodym derivative $\frac{d (\Phi _t)_*\vec {\mu }_s}{d \vec {\mu }_s}$. While this formula has not been used in the context of wave equations, for other deterministic models, it allowed to improve quasi-invariance results. See [10, 14, 18, 20, 22, 23, 32] for some examples. However, in the stochastic case, we do not have such an explicit formula for the density, as we cannot solve the Fokker-Plank equation (5.5) explicitly.

#### Remark 1.6

A very recent result by Hairer-Kusuoka-Nagoji [30] shows the reverse of Theorem 1.1 for a wide class of parabolic equations, i.e. that under certain assumptions, the invariant measure for singular parabolic SPDEs is singular with respect to the law of the stationary linear solution. Interestingly, the main assumption of their result, i.e. the fact that $\Phi _t - \Phi _t^{\text {lin}}$ does not belong to the Cameron-Martin space for $\vec {\mu _s}$, also applies to equation (1.1). Nevertheless, the result of this paper shows that the measure is quasi-invariant. The discrepancy between these results is due to the following.

- The result in [30] heavily relies on the fact that the nonlinearity of the equation is not an $L^1_{\text {loc}}$ function when computed in the linear solution, and
- The dispersive/Hamiltonian structure of the wave equation introduces further cancellations that are not present in the parabolic case.

## Preliminaries

**Notations.** For a function $f: \mathbb {T}^2 \sim [0,1]^2 \rightarrow \mathbb {C}$, its Fourier series is given by $\widehat{f}: \mathbb {Z}^2 \rightarrow \mathbb {C}$, where

$$\begin{aligned} \widehat{f} (n) := \int _{\mathbb {T}^2} f(x) e^{-2\pi i n \cdot x} d x \quad \text {and} \quad f(x) := \sum _{n\in \mathbb {Z}^2} \widehat{f}(n) e^{2\pi i n \cdot x}. \end{aligned}$$

Moreover, for a function $F: \mathbb {C}^2 \rightarrow \mathbb {C}$, the expression $F(\nabla )u$ will denote the distribution with Fourier coefficients given by

$$\begin{aligned} \widehat{F(\nabla ) u}(n) := F(2 \pi i n) \widehat{u} (n). \end{aligned}$$

For $x \in \mathbb {R}^2$, we denote $\langle x \rangle := \big (1 + |x|^2\big )^\frac{1}{2}$. Given $N\in \mathbb {N}_{0}:=\mathbb {N}\cup \{0\}$, we define $\Pi _{\le N}$ as the sharp projection onto frequencies in the square $[-N,N]^{2}$, that is,

$$\begin{aligned} \Pi _{\le N}u(x)= \frac{1}{(2\pi )^{2}} \sum _{\begin{array}{c} n\in \mathbb {Z}^{2}\\ |n|_{\infty }\le N \end{array}} \widehat{u}(n)e^{in\cdot x}, \end{aligned}$$

where for $n=(n_1,n_2)$, $|n|_{\infty }:= \max _{j}|n_j|$, and similarly, when ${\textbf{u}}$ is vector valued,

$$\begin{aligned} \Pi _{\le N}{\textbf{u}}(x)= ( \Pi _{\le N} \pi _1 {\textbf{u}}(x), \Pi _{\le N} \pi _2 {\textbf{u}}(x)). \end{aligned}$$

We also define $\Pi _{>N}:=\text {Id}-\Pi _{\le N}$ and for $N=-1$, extend these projections to $\Pi _{\le -1}=0$ and $\Pi _{>-1}=\text {Id}$. As $\Pi _{\le N}$ is the Fourier restriction to a cube with sides of length $2N+1$, we can rewrite $\Pi _{\le N}$ as the composition of the Hilbert transform on each of the variables. Thus, from the boundedness of the Hilbert transform on $L^p(\mathbb {T})$ for $1< p < \infty$, we obtain

$$\begin{aligned} \Vert \Pi _{\le N} u\Vert _{L^p(\mathbb {T}^2)} \lesssim \Vert u \Vert _{L^p(\mathbb {T}^2)}, \end{aligned}$$

uniformly in the parameter *N* and for $1< p<\infty$. When $N=+\infty$, we set $\Pi _{\le +\infty }:=\text {Id}$.

Given a vector ${\textbf{u}}=(u,v)^{\top }$, we use $\pi _1$ and $\pi _2$ to denote the projection onto the first and second components of ${\textbf{u}}$, respectively. Namely, $\pi _1 {\textbf{u}}=u$ and $\pi _2 {\textbf{u}}=v$.

### Function spaces

We define the Sobolev spaces $W^{\alpha ,p}$ and $\mathcal {W}^{\alpha ,p}:=W^{\alpha ,p}\times W^{\alpha -1,p}$ via the norms

$$\begin{aligned} \Vert u\Vert _{W^{\alpha ,p}}:= \Vert \langle \nabla \rangle ^\alpha u\Vert _{L^p} \quad \text {and} \quad \Vert {\textbf{u}}\Vert _{\mathcal {W}^{\alpha , p}}^p := \Vert \langle \nabla \rangle ^\alpha u\Vert _{L^p}^p + \Vert \langle \nabla \rangle ^{\alpha -1}v\Vert _{L^p}^p, \end{aligned}$$

with the usual modification when $p=\infty$. Moreover, when $p = 2$, we write $\mathcal {H}^\alpha := \mathcal {W}^{\alpha ,2}$.

We define Littlewood-Payley projections as follows. Let $\phi :\mathbb {R}\rightarrow [0,1]$ be a smooth bump function supported on $[-\frac{8}{5}, \frac{8}{5}]$ and $\phi =1$ on $[-\frac{5}{4}, \frac{5}{4}]$. For $\xi \in \mathbb {R}^2$, we set $\phi _{0}(\xi ):=\phi (|\xi |)$ and $\phi _{j}(\xi ) = \phi _{0}(2^{-j}\xi )-\phi _0 (2^{-j+1}\xi )$ for $j\in \mathbb {N}$. For $j\in \mathbb {N}_{0}$, we define the Littlewood-Payley projector $P_{N}$ as the Fourier multiplier operator with symbol

$$\begin{aligned} \varphi _{N}(\xi ) = {\phi _{N}(\xi )}, \end{aligned}$$

where $N:=2^{j}$. This gives a decomposition of a distribution

$$\begin{aligned} f = \sum _{N\in 2^{\mathbb {N}_0}} P_N f. \end{aligned}$$

We will also denote $P_{\le N} = \sum _{M \le N} P_N$. We recall the definition of the Besov spaces $B^{\alpha }_{p,q}(\mathbb {T}^2)$ which are defined by norm:

2.1 $$\begin{aligned} \Vert u\Vert _{B^{\alpha }_{p,q}} = \big \Vert N^{\alpha } \Vert P_{N} u\Vert _{L^{p}_{x}} \big \Vert _{\ell ^{q}_{N}}. \end{aligned}$$

We have $B^{\alpha }_{2,2}=H^{\alpha }$ for any $\alpha \in \mathbb {R}$, and we define the Besov-Hölder spaces $C^{\alpha }:=B^{\alpha }_{\infty ,\infty }$. We also define vector versions of these spaces as $\mathcal {C}^{\alpha }:=C^{\alpha }\times C^{\alpha -1}$ with the norm

$$\begin{aligned} \Vert {\textbf{u}}\Vert _{\mathcal {C}^{\alpha }} = \Vert \pi _1 {\textbf{u}}\Vert _{C^{\alpha }}+\Vert \pi _2 {\textbf{u}}\Vert _{C^{\alpha -1}}. \end{aligned}$$

The Besov spaces have the following properties. See for instance [3].

#### Lemma 2.1

The following estimates hold.

(i) (interpolation) For $0<s_1<s_2$ and $\theta =\frac{s_1}{s_2}$, it holds that

2.2 $$\begin{aligned} \Vert u\Vert _{H^{s_1}} \lesssim \Vert u\Vert _{H^{s_2}}^{\theta }\Vert u\Vert _{L^2}^{1-\theta }. \end{aligned}$$

(ii) (embeddings) Let $s_1,s_2\in \mathbb {R}$ and $p_1,p_2, q_1,q_2\in [1,\infty ]$. Then,

2.3 $$\begin{aligned} \begin{aligned} \Vert u\Vert _{B^{s_1}_{p_1,q_1}}&\lesssim \Vert u\Vert _{B^{s_2}_{p_2,q_2}} \quad \text {for} \,\, s_1 \le s_2, p_1\le p_2,\,\, \text {and} \,\, q_1\ge q_2, \\ \Vert u\Vert _{B^{s_1}_{p_1,q_1}}&\lesssim \Vert u\Vert _{B^{s_2}_{p_1,\infty }} \quad \text {for} \,\, s_1<s_2,\\ \Vert u\Vert _{B^{0}_{p_1,\infty }}&\lesssim \Vert u\Vert _{L^{p_1}} \lesssim \Vert u\Vert _{B^{0}_{p_1,1}}. \end{aligned} \end{aligned}$$

(iii) (algebra property) For any $\sigma >0$,

2.4 $$\begin{aligned} \Vert uv \Vert _{C^{\sigma }} \lesssim \Vert u\Vert _{C^\sigma }\Vert v\Vert _{C^{\sigma }}. \end{aligned}$$

(iv) (duality) Let $\sigma \in \mathbb {R}$ and $p,p',q,q'\in [1,\infty ]$ such that $\frac{1}{p}+\frac{1}{p'}=\frac{1}{q}+\frac{1}{q'}=1$. Then,

2.5 $$\begin{aligned} \bigg |\int _{\mathbb {T}^2}uv dx \bigg | \le \Vert u\Vert _{B^{\sigma }_{p,q}}\Vert v\Vert _{B^{-\sigma }_{p',q'}}. \end{aligned}$$

(iv) (fractional Leibniz rule) Let $p,p_1,p_2,p_3,p_4\in [1,\infty ]$ such that $\frac{1}{p_1}+\frac{1}{p_2}=\frac{1}{p_3}+\frac{1}{p_4}=\frac{1}{p}$. Then, for all $\sigma >0$, we have

2.6 $$\begin{aligned} \Vert uv\Vert _{B^{\sigma }_{p,q}} \lesssim \Vert u\Vert _{B^{\sigma }_{p_1,q}}\Vert v\Vert _{L^{p_2}} + \Vert u\Vert _{L^{p_3}}\Vert v\Vert _{B^{\sigma }_{p_4,q}}. \end{aligned}$$

(iv) (Sobolev embedding) Let $1\le p_2\le p_1\le \infty$, $q\in [1,\infty ]$, and $s_2 \ge s_1+ 2(\frac{1}{p_2}-\frac{1}{p_1})$. Then,

2.7 $$\begin{aligned} \Vert u\Vert _{B^{s_1}_{p_1,q}} \lesssim \Vert u\Vert _{B^{s_2}_{p_2,q}}. \end{aligned}$$

Given two functions *f* and *g* on $\mathbb {T}^2$, we can expand the product *fg* as2.8We will use the shorthand $N\ll M$ for $N<\frac{1}{2}M$ and $N\sim M$ for $\frac{1}{2}M \le N \le 2M$.

#### Lemma 2.2

Let $s_1, s_2 \in \mathbb {R}$ and $1 \le p, p_1, p_2, q \le \infty$ such that $\frac{1}{p} = \frac{1}{p_1} + \frac{1}{p_2}$. Then, we have2.9When $s_1 < 0$, we have2.10When $s_1 + s_2 > 0$, we have2.11In particular, if $s_1<0<s_2$ and $s_1+s_2>0$, the mapping $(f,g)\rightarrow fg$ extends to a continuous bilinear map from $B^{s_1}_{p_1,q}\times B^{s_2}_{p_2,q}$ to $B^{s_1+s_2}_{p,q}$.

See [3] for proofs in the non-periodic case. We then have some useful consequences.

#### Lemma 2.3

Let $0<\alpha <s$ and $0<\delta <2(1-s+\alpha )$. Then,

2.12 $$\begin{aligned} \Vert fg\Vert _{H^{-s}} \lesssim \Vert f\Vert _{H^{-s+\alpha }}\Vert g\Vert _{C^{s-\alpha +\delta }}, \end{aligned}$$

2.13 $$\begin{aligned} \Vert fg\Vert _{H^{-s+\alpha }} \lesssim \Vert f\Vert _{H^{s-\alpha +\delta }}\Vert g\Vert _{C^{-s+\alpha }}. \end{aligned}$$

#### Proof

First assume that $0<s<1$ and consider (2.12). Let $p=\frac{2}{1+s+\frac{1}{2}\delta }$ with $0<\frac{1}{2}\delta <1-s$ sufficiently small so that $p>1$. By Sobolev embedding and Lemma 2.2, we have

2.14 $$\begin{aligned} \Vert fg\Vert _{H^{-s}} \lesssim \Vert fg\Vert _{B^{\frac{1}{2}\delta }_{p,2}} \lesssim \Vert f\Vert _{H^{-s+\alpha }}\Vert g\Vert _{B_{\infty ,2}^{s-\alpha +\frac{1}{2}\delta }} \lesssim \Vert f\Vert _{H^{-s+\alpha }}\Vert g\Vert _{C^{s-\alpha +\delta }}. \end{aligned}$$

For (2.13), with $p=\frac{2}{1+\frac{1}{2}\delta +s-\alpha }$ and $0<\delta <2(1-s+\alpha )$, when $0<s<1$:

$$\begin{aligned} \Vert fg\Vert _{H^{-s+\alpha }}\lesssim \Vert fg\Vert _{B^{\frac{1}{2} \delta }_{p,2}} \lesssim \Vert f\Vert _{H^{s-\alpha +\delta }} \Vert g\Vert _{B^{-s+\alpha -\frac{1}{2} \delta }_{\infty ,2}} \lesssim \Vert f\Vert _{H^{s-\alpha +\delta }}\Vert g\Vert _{C^{-s+\alpha }}. \end{aligned}$$

If $s\ge 1$, then we instead first use the embedding $B^{\frac{1}{2}\delta }_{1,2}\subset H^{-s}$ for both (2.12) and (2.13). $\square$

Let *S*(*t*) be the linear propagator for the (damped) wave equation, which is given by the formula

2.15 $$\begin{aligned} e^{-\frac{t}{2}} \begin{pmatrix}\cos \big (t[ \nabla ]\big ) + \frac{1}{2}\frac{\sin (t[ \nabla ])}{[ \nabla ]} & \frac{\sin (t\langle [ \nabla ] \rangle )}{[ \nabla ]} \\ -\big ([ \nabla ]+\frac{1}{4[ \nabla ]}\big )\sin (t[ \nabla ]) &  \cos (t[ \nabla ]) -\frac{1}{2}\frac{\sin (t[ \nabla ])}{[ \nabla ]} \end{pmatrix}, \end{aligned}$$

where $[ \nabla ]:= (\frac{3}{4} -\Delta )^{\frac{1}{2}}$. With this definition, it holds that for every $t \in \mathbb {R}$,

2.16 $$\begin{aligned} \Vert S(t){\textbf{u}}\Vert _{\mathcal {H}^{\alpha }} \lesssim e^{-\frac{t}{2}}\Vert {\textbf{u}}\Vert _{\mathcal {H}^{\alpha }}. \end{aligned}$$

Following [19], for $0<\alpha < 1$, we define the space

$$\begin{aligned} \overline{X}^{\alpha }:=\{ {\textbf{u}}\in \mathcal {H}^\alpha \, : \, S(t){\textbf{u}}\in C([0,+\infty ); \mathcal {W}^{\alpha , \frac{2}{\alpha }}), \, \Vert S(t){\textbf{u}}\Vert _{\mathcal {W}^{\alpha ,\frac{2}{\alpha }}}\lesssim e^{-\frac{t}{8}} \}, \end{aligned}$$

with the norm

2.17 $$\begin{aligned} \Vert {\textbf{u}}\Vert _{ X^\alpha }:=\sup _{t\ge 0} \, e^{\frac{t}{8}}\Vert S(t){\textbf{u}}\Vert _{\mathcal {W}^{\alpha ,\frac{2}{\alpha }}}. \end{aligned}$$

We define $X^{\alpha }$ to be the closure of trigonometric polynomials in $\overline{X}^{\alpha }$ under the norm $\Vert \cdot \Vert _{ X^\alpha }$, which makes $( X^\alpha , \Vert \cdot \Vert _{ X^\alpha })$ into a Polish space. Note that for any $t\ge 0$,

2.18 $$\begin{aligned} \Vert S(t)\Vert _{X^{\alpha }\mapsto X^\alpha }\le e^{-\frac{t}{8}}. \end{aligned}$$

Moreover the following embeddings hold (see [19]):

$$\begin{aligned} \mathcal {H}^{1} \hookrightarrow X^{\alpha } \hookrightarrow \mathcal {W}^{0,6}. \end{aligned}$$

When $\alpha \ge 1$, we simply define $X^{\alpha }:=\mathcal {H}^{\alpha }$, and, when $\alpha >1$, the embedding $\mathcal {H}^{\alpha }\hookrightarrow L^{\infty }$ implies that $X^{\alpha }$ is an algebra.

Similar to the spaces $\overline{X}^{\alpha }$ and $X^\alpha$, given $\alpha >0$, we let

$$\begin{aligned} \overline{Z}^{\alpha }:=\{ {\textbf{u}}\in \mathcal {H}^{\alpha } \, :\, S(t){\textbf{u}}\in C([0,+\infty ); \mathcal {C}^{\alpha }), \,\, \Vert S(t){\textbf{u}}\Vert _{\mathcal {C}^{\alpha }}\lesssim e^{-\frac{t}{8}}\, \}, \end{aligned}$$

and define $Z^{\alpha }$ to be the closure of trigonometric polynomials in $\overline{Z}^{\alpha }$ under the norm

2.19 $$\begin{aligned} \Vert {\textbf{u}}\Vert _{Z^{\alpha }}=\sup _{t\ge 0} e^{\frac{t}{8}}\Vert S(t){\textbf{u}}\Vert _{\mathcal {C}^{\alpha }}. \end{aligned}$$

Now, $(Z^{\alpha },\Vert \cdot \Vert _{Z^{\alpha }})$ is a Polish space (see [52, Lemma 1.2]) and $Z^{\alpha }\hookrightarrow X^{\alpha }$.

### A commutator estimate

Our main goal in this subsection is to give a proof of the following commutator estimate.

#### Proposition 2.4

For any $s>0$ and $0<\varepsilon <\frac{1}{4}\min (s,1)$, it holds that

2.20 $$\begin{aligned} \Vert \langle \nabla \rangle ^{s}(u^3)-3u^2 \langle \nabla \rangle ^{s} u\Vert _{L^{2}(\mathbb {T}^2)} \lesssim \Vert u\Vert _{C^{s-\varepsilon }(\mathbb {T}^2)}^{3}. \end{aligned}$$

Whilst the estimate (2.20) is far from optimal, it will be sufficient for our purposes as we only require the regularities on the right-hand side of (2.20) to be strictly less than *s*. We need two preliminary results before we can give a proof of (2.20).

#### Lemma 2.5

For any $s>0$ and $0<\varepsilon <\frac{1}{2} s$, it holds that2.21

#### Proof

We have

$$\begin{aligned} u^3&= \sum _{N} \big \{(P_{\le 2N}u)^3-(P_{\le N}u)^3 \big \}= \sum _{N} P_{N}u \big [ (P_{\le 2N}u)^2+(P_{\le 2N}u)(P_{\le N}u)+(P_{\le N}u)^2\big ], \end{aligned}$$

so that2.222.232.24We provide details only for estimating (2.22) as the (2.23) and (2.24) follow from similar ideas exploiting the existence of at least two similar and large frequencies. We write

2.25 $$\begin{aligned} \begin{aligned} (P_{\le 2N}u)^{2}-P_{\le \frac{1}{2}N}(u^2)&=\sum _{N_1,N_2} P_{>\frac{1}{2}N}[ P_{\le 2N}P_{N_1}u \cdot P_{\le 2N}P_{N_2}u] \\&+ P_{\le \frac{1}{2}N} \big [ P_{\le 2N}P_{N_1}u\cdot P_{\le 2N}P_{N_2}u - P_{N_1}u \cdot P_{N_2}u\big ]. \end{aligned} \end{aligned}$$

Let $M\in 2^{\mathbb {N}_0}$. For the first piece on the right-hand side of (2.25), we have

2.26 $$\begin{aligned} \begin{aligned} \bigg \Vert \sum _{N,N_1,N_2}&P_{M} \big [ P_{N}u \cdot P_{>\frac{1}{2}N}[ P_{\le 2N}P_{N_1}u \cdot P_{\le 2N}P_{N_2}u]\big ]\bigg \Vert _{L^2} \\&\lesssim \sum _{\begin{array}{c} N,N_1,N_2\\ N_1 \sim N \gtrsim M \end{array}} \Vert P_{N}u \cdot P_{>\frac{1}{2}N}[ P_{\le 2N}P_{N_1}u \cdot P_{\le 2N}P_{N_2}u]\big ]\Vert _{L^2} \\&\lesssim \sum _{\begin{array}{c} N,N_1,N_2\\ N_1 \sim N \gtrsim M \end{array}} \Vert P_{N}u\Vert _{L^{\infty }} \Vert P_{N_1}u\Vert _{L^{2}}\Vert P_{N_2}u\Vert _{L^{\infty }} \\&\lesssim \bigg ( \sum _{\begin{array}{c} N,N_1,N_2\\ N_1 \sim N \gtrsim M \end{array}} (NN_1 N_2)^{-(s-\varepsilon )} \bigg ) \Vert u\Vert ^{3}_{C^{s-\varepsilon }} \\&\lesssim M^{-2(s-\varepsilon )}\Vert u\Vert ^{3}_{C^{s-\varepsilon }}. \end{aligned} \end{aligned}$$

Using the characterisation

$$\begin{aligned} \Vert f\Vert _{H^{s}}^2 \sim \sum _{M} M^{2s} \Vert P_{M}f\Vert _{L^{2}}^{2}, \end{aligned}$$

we see that this contribution is controlled provided that $2s-4(s-\varepsilon )<0$, enforcing $\varepsilon <\frac{1}{2}s$. For the second piece on the right hand side of (2.25), we further write it as

$$\begin{aligned} P_{\le \frac{1}{2}N}&\big [ P_{\le 2N}P_{N_1}u\cdot P_{\le 2N}P_{N_2}u - P_{N_1}u \cdot P_{N_2}u\big ] \\&= P_{\le \frac{1}{2}N} \big [ P_{\le 2N}P_{N_1}u \cdot P_{>2N}P_{N_2}u - (P_{>2N}P_{N_1}u)(P_{N_2}u)\big ] \end{aligned}$$

Again, we have at least that $\max (N_1,N_2)\gtrsim N$ in each of these terms, and we can proceed similar to (2.26) provided that $\varepsilon <\frac{1}{2}s$. This completes the proof. $\square$

#### Lemma 2.6

Given $s>0$ and $0<\varepsilon <\frac{1}{2}s$, there exists a multilinear operator *R* such that2.27and *R* satisfies

$$\begin{aligned} \Vert R(f,g)\Vert _{L^{2}} \lesssim \Vert f\Vert _{C^{s-\varepsilon }}\Vert g\Vert _{B^{s-\varepsilon }_{2,\infty }}. \end{aligned}$$

Lemma 2.6 is well known in the case of $\mathbb {R}^2$; see the more general result in [3, Theorem 2.92]. For an argument in the periodic setting, we refer to [46, Lemma 13].

#### Proof of Proposition 2.4

We begin by writing

2.28 $$\begin{aligned} \langle \nabla \rangle ^{s}(u^3) -3u^2 \langle \nabla \rangle ^{s} u =&|\nabla |^{s}(u^3) -3u^2 |\nabla |^{s} u \end{aligned}$$

2.29 $$\begin{aligned}&+ (\langle \nabla \rangle ^{s}-|\nabla |^{s})(u^3) - 3u^2 ( \langle \nabla \rangle ^{s}-|\nabla |^{s})(u). \end{aligned}$$

By the mean-value theorem,

2.30 $$\begin{aligned} |\langle n \rangle ^{s}-|n|^{s}| \lesssim \langle n \rangle ^{s-1} \quad \text {for all} \quad n\in \mathbb {Z}^{2}. \end{aligned}$$

If $s\le 1$, then (2.30) implies

2.31 $$\begin{aligned} \Vert (\langle \nabla \rangle ^{s}-|\nabla |^{s})(u^3) \Vert _{L^2} \lesssim \Vert u\Vert _{L^6}^{3}. \end{aligned}$$

Otherwise, if $s>1$, (2.6) implies

2.32 $$\begin{aligned} \Vert (\langle \nabla \rangle ^{s}-|\nabla |^{s})(u^3) \Vert _{L^2} \lesssim \Vert u^3\Vert _{H^{s-1}}\lesssim \Vert u\Vert _{L^{\infty }}^{2} \Vert u\Vert _{H^{s-1+\varepsilon }} \end{aligned}$$

for any $\varepsilon >0$. We also have by (2.30) that

2.33 $$\begin{aligned} \Vert u^2 ( \langle \nabla \rangle ^{s}-|\nabla |^{s})(u)\Vert _{L^2} \lesssim \Vert u\Vert _{L^{\infty }}^{2}\Vert u\Vert _{H^{s-1}}. \end{aligned}$$

Now (2.31), (2.32), and (2.33) give

$$\begin{aligned} (2.29) \lesssim \Vert u\Vert _{C^{s-\varepsilon }}^{3}. \end{aligned}$$

For (2.28), we write2.342.352.36First, we haveUsing (2.11) and (2.4), we haveWe obtain the same bound for  by using (2.10). Thus,

$$\begin{aligned} (2.36) \lesssim \Vert u\Vert _{C^{s-\varepsilon }}^{3}. \end{aligned}$$

For (2.34), we use (2.21). It remains to control (2.35) for which we use Lemma 2.6. For the remainder term, we obtain the bound $\lesssim \Vert u\Vert _{C^{s-\varepsilon }}^{3}$, after using (2.4). For the first term, we fix $j=1,2$, and use (2.9) and (2.4),Now, if $s>1$, taking $\varepsilon <\frac{1}{2}$ we may sum over the dyadics. If $s\le 1$, we need $\varepsilon <\frac{1}{2}s$. $\square$

### The white noise and stochastic convolution

The space-time white noise $\xi$ is the unique (in law) random distribution such that $\{ \langle \xi ,\phi \rangle \}_{\phi \in L^2(\mathbb {R}\times \mathbb {T}^2)}$ is a family of centred Gaussian random variables on a probability space $(\Omega , \mathcal {F}, \mathbb {P})$ with the property that

2.37 $$\begin{aligned} \mathbb {E}\big [ \langle \xi , \phi \rangle \langle \xi , \psi \rangle \big ]=\langle \phi , \psi \rangle _{L^2(\mathbb {R}\times \mathbb {T}^2)} \end{aligned}$$

for any $\phi , \psi \in C^\infty _c(\mathbb {R}\times \mathbb {T}^2)$. For $t\ge 0$, we set

$$\begin{aligned} \tilde{\mathcal {F}}_{t}=\sigma \big ( \big \{ \langle \xi ,\phi \rangle \, : \, \phi \vert _{(t,+\infty )\times \mathbb {T}^2}\equiv 0, \, \phi \in L^2(\mathbb {R}\times \mathbb {T}^2)\big \}\big ) \end{aligned}$$

and we denote by $\{\mathcal {F}_{t}\}_{t\ge 0}$ the usual augmentation of the filtration $\{ \tilde{\mathcal {F}}_{t}\}_{t\ge 0}$ (see [48, p. 45]).

We define the stochastic convolution  in a pathwise manner as the space-time distribution satisfying2.38for all test functions $\phi =( \phi _1, \phi _2)^{\top }:\mathbb {T}^2 \rightarrow \mathbb {R}$. Here, we recall that *S*(*t*) is given by (2.15). For smooth $\xi$, the definition (2.38) corresponds exactly to2.39It enjoys the following regularity property.

#### Lemma 2.7

Let $0<\alpha <s$ and $N\in 2^{\mathbb {N}_0}\cup \{\infty \}$. Then,  almost surely. Moreover, for every $T>0$,2.40where $1\le p<\infty$.

Before we give the proof, we first recall a quantitative version of the Kolmogorov continuity criterion, which is a slight modification of [31, Lemma 2.4].

#### Lemma 2.8

Given $\varepsilon >0$, $j\in \mathbb {N}_0$, $\gamma \in (0,1)$, $1\le p<\infty$, a Banach space *X*, and a process $F:[j,j+1]\rightarrow X$ that is almost surely continuous,

2.41 $$\begin{aligned} \mathbb {E}\big [ \Vert F\Vert _{C^{\gamma }([j,j+1];X)}^{p} \big ] \lesssim _{\gamma ,\varepsilon } \mathbb {E}[ \Vert F(j)\Vert _{X}^{p}] + \sup _{j\le t'<t\le j+1} \mathbb {E}\bigg [ \frac{ \Vert F(t)-F(t')\Vert _{X}^{p} }{|t-t'|^{1+\gamma p +\varepsilon }} \bigg ]. \end{aligned}$$

#### Proof of Lemma 2.7

The almost sure properties and the moment bound (2.40) when $N=+\infty$ were proved in [19, Proposition 2.7 and 2.8]. Note that this does not automatically imply (2.40), since the operator $\Pi _{\le N}$ is not bounded on $\mathcal {C}^{\alpha }$. However, the estimate immediately follows from the arguments in [19, Proposition 2.8], so here we highlight the main modifications to the argument. For (2.40), a telescoping series argument and Minkowski’s inequality shows that it suffices to prove that2.422.43for some $\theta >0$, and every $M\in 2^{\mathbb {N}_0}$.

For shorthand, we define . To control the supremum over $t\in [0,T]$ in (2.43), we decompose [0, *T*] into unit intervals and then apply Lemma 2.8 on each interval. We only discuss the first term on the right-hand side of (2.41). The estimates required for the time-differences term in (2.41) follow similarly using the strategy below and the estimates in the proof of [19, Proposition 2.7], up to the modification we give below. Fix $t\in (0,T]$. Recalling the definition of the $Z^{\alpha }$-norm in (2.19), for finite $p>\frac{2}{s-\alpha }$, Minkowski’s inequality impliesTo control the supremum over $\tau$, we again need to use Lemma 2.8. We again only discuss the contribution from the first term from (2.41). By Sobolev embedding and the Gaussianity of $\xi$, we havewhere $\phi _{n} = ( e_n, \langle n \rangle ^{-1}e_n)^{\top }$. Using (2.38) and the frequency localisations from the difference in , which forces $|n|_{\infty }\sim M$, we bound this by

$$\begin{aligned} e^{-\frac{T'}{2}}\bigg ( \sum _{|n|_{\infty }\sim M} \langle n \rangle ^{2\alpha +\frac{4}{p}-2s-2} \bigg )^{\frac{1}{2}} \lesssim e^{-\frac{T'}{2}} M^{-(s-\alpha -\frac{2}{p})}, \end{aligned}$$

which gives (2.43), as long as $p>\frac{2}{s-\alpha }$. Finally, (2.42) follows similarly. $\square$

## The flow for (1.1)

### Review of well-posedness

In this section, we recall elements from [19] of the notion of solution and associated well-posedness for the equation (1.1). We view (1.1) in the following integral formulation:

3.1 $$\begin{aligned} {\textbf{u}}= S(t){\textbf{u}}_0 +\int _{0}^{t}S(t-t') \begin{pmatrix} 0 \\ {{{\sqrt{2}}}} \langle \nabla \rangle ^{-s}\xi (t') \end{pmatrix} dt' -\int _{0}^{t}S(t-t')\begin{pmatrix} 0 \\ (\pi _{1}{\textbf{u}}(t'))^{3} \end{pmatrix}dt', \end{aligned}$$

where $\pi _1$ denotes the projection to the first coordinate. Motivated by (3.1) and these considerations about the stochastic convolution (2.39), we define a solution of (1.1) in terms of the first-order expansion [6, 12, 35]:3.2where ${\textbf{w}}=(w, \partial _tw)^{\top }$ solves the equation3.3We note that the equation (3.3) is chosen to be the mild formulation of the differential equationwhich we obtain by inserting the ansatz (3.2) into the equation (1.1). We recall the well-posedness statement for (3.3) below in Proposition 3.1.

In order to make the arguments in this paper rigorous, we require approximating finite dimensional versions of (1.1), which we describe now. Given $N\in 2^{\mathbb {N}_0}$, we define the truncated system of (1.3) by

3.4 $$\begin{aligned} {\left\{ \begin{array}{ll} \partial _t \begin{pmatrix}{u^N}\\ {v^N}\end{pmatrix} & = -\begin{pmatrix} 0 &  -1 \\ 1-\Delta &  1 \end{pmatrix} \begin{pmatrix} u^N \\ v^N \end{pmatrix} - \Pi _{\le N} \begin{pmatrix}{0}\\ {(\Pi _{\le N}u^N)^3}\end{pmatrix} + \begin{pmatrix} {0}\\ {\sqrt{2} \langle \nabla \rangle ^{-s}\xi }\end{pmatrix} \\ \begin{pmatrix} u^N \\ v^N \end{pmatrix}(0) &  = \begin{pmatrix} u_0 \\ u_1 \end{pmatrix}. \end{array}\right. } \end{aligned}$$

Solutions to (3.4) naturally decompose as:

$$\begin{aligned} {\textbf{u}}^{N}(t) = \Pi _{\le N} {\textbf{u}}^N (t) + \Pi _{>N}{\textbf{u}}^N(t)= {\textbf{u}}_{N} (t)+{\textbf{u}}^{\perp }_{N}(t), \end{aligned}$$

where the low-frequency part ${\textbf{u}}_N (t) =: (u_N, v_N)$ solves

3.5 $$\begin{aligned} {\left\{ \begin{array}{ll} \partial _t \begin{pmatrix} {u_N}\\ {v_N} \end{pmatrix} & = -\begin{pmatrix} 0 &  -1 \\ 1-\Delta &  1 \end{pmatrix} \begin{pmatrix} u_N \\ v_N \end{pmatrix} - \Pi _{\le N} \begin{pmatrix} {0}\\ {u_N^3} \end{pmatrix}+ \begin{pmatrix} {0}\\ {\sqrt{2} \langle \nabla \rangle ^{-s} \Pi _{\le N}\xi }\end{pmatrix} \\ \begin{pmatrix} u_N \\ v_N \end{pmatrix}(0) &  = \begin{pmatrix} \Pi _{\le N} u_0 \\ \Pi _{\le N}u_1 \end{pmatrix}. \end{array}\right. } \end{aligned}$$

and the high-frequency part ${\textbf{u}}^{\perp }_{N} =:(u_{N}^{\perp }, v_{N}^{\perp })$ solves the linear damped stochastic wave equation:

3.6 $$\begin{aligned} {\left\{ \begin{array}{ll} \partial _t \begin{pmatrix} {u^{\perp }_N}\\ {v^{\perp }_N} \end{pmatrix} & = -\begin{pmatrix} 0 &  -1 \\ 1-\Delta &  1 \end{pmatrix} \begin{pmatrix} u^{\perp }_N \\ v^{\perp }_N \end{pmatrix} + \begin{pmatrix} {0}\\ {\sqrt{2} \langle \nabla \rangle ^{-s} \Pi _{>N}\xi }\end{pmatrix} \\ \begin{pmatrix} u^{\perp }_N \\ v^{\perp }_N \end{pmatrix}(0) &  = \begin{pmatrix} \Pi _{> N} u_0 \\ \Pi _{>N}u_1 \end{pmatrix}. \end{array}\right. } \end{aligned}$$

We write solutions ${\textbf{u}}_{N}$ to (3.5) as3.7where3.8is the solution map for the linear stochastic damped NLW, and ${\textbf{w}}_N$ solves3.9We will occasionally use the fact that $\Pi _{\le N}\Phi ^{\text {lin}}_{t}({\textbf{u}}_0,\xi ) = \Phi ^{\text {lin}}_{t}(\Pi _{\le N}{\textbf{u}}_0,\Pi _{\le N}\xi )$ without explicit reference. We have the following global well-posedness results for (1.1) and (3.9).

#### Proposition 3.1

[19, Section 3] Let $N\in 2^{\mathbb {N}_0}$, $s>0$, $0<\alpha <s$, $T>0$, ${\textbf{u}}_0 \in X^\alpha$, and ${\textbf{w}}_0\in \mathcal {H}^1$. Then, the equations3.103.11are almost surely globally-well posed in $\mathcal {H}^{1}(\mathbb {T}^2)$. Moreover, the following hold true:

(i) With ${\textbf{w}}_N$ denoting the solution to (3.11) with ${\textbf{w}}_0\equiv 0$, for every $N\in 2^{\mathbb {N}_0}\cup \{\infty \}$ and for $0<\alpha <s\wedge 1$, it holds that3.12(ii) For every $T<\infty$, we have

$$\begin{aligned} \lim _{N\rightarrow \infty } \Vert {\textbf{w}}-{\textbf{w}}_N\Vert _{C([0,T];\mathcal {H}^1)}=0 \quad a.s. \end{aligned}$$

The existence and uniqueness of ${\textbf{w}}$ and ${\textbf{w}}_N$ is guaranteed by an application of Banach fixed point theorem, which uses only the fractional Leibniz rule and Sobolev embedding. The global bound (3.12) follows from a Gronwall argument on an energy quantity resembling that of the Hamiltonian for the undamped, non-stochastic NLW; see [19, (3.12)]. The a priori bound (3.12) is only of interest when $s<1$. When $s\ge 1$, we still have global well-posedness for ${\textbf{w}}$ by a Gronwall argument with the energy

3.13 $$\begin{aligned} \begin{aligned} H_{N}(u,v)&:= H(\Pi _{\le N} u, \Pi _{\le N}v),\\ H(u,v)&:= \frac{1}{2}\int _{\mathbb {T}^{2}}u^2+| \nabla u|^{2} +v^2 dx+ \frac{1}{4}\int _{\mathbb {T}^2} u^4 dx. \end{aligned} \end{aligned}$$

We omit the details. See for example [8].

We remark that by regularity counting arguments, we expect ${\textbf{w}}$ to be more regular than $\mathcal {H}^{1}(\mathbb {T}^2)$ and to belong to $\mathcal {H}^{1+\alpha }(\mathbb {T}^2)$. This additional regularity can be justified a posteriori from Proposition 3.1, and we will make use of it later; see Lemma 4.6.

Recall that for ${\textbf{u}}_0\in X^\alpha$, and $N\in 2^{\mathbb {N}_0}$, we have

3.14 $$\begin{aligned} \Phi _{t}^{N}({\textbf{u}}_0; \xi ):={\textbf{u}}^{N}(t) = {\textbf{u}}_{N}(t) + {\textbf{u}}^{\perp }_{N}(t), \end{aligned}$$

where ${\textbf{u}}_N$ has the decomposition (3.7) and ${\textbf{u}}^{\perp }_{N}(t)$ solves the linear equation (3.6). When $N=\infty$, we write3.15where ${\textbf{w}}$ solves (3.10). We have the following convergence statement for the nonlinear flows.

#### Lemma 3.2

Let $s>0$, $0<\alpha <s$, ${\textbf{u}}_0\in X^{\alpha }$, and $T>0$. Then, $\Phi _{t}^{N}({\textbf{u}}_0,\xi )$ converges to $\Phi _{t}({\textbf{u}}_0,\xi )$ almost surely in $C([0,T];\mathcal {H}^{\alpha }(\mathbb {T}^2))$ as $N\rightarrow \infty$.

#### Proof

When $\alpha <1$, this is immediate from (3.14), (3.15), and Proposition 3.1 (ii). When $\alpha \ge 1$, we can estimate the difference ${\textbf{w}}_N -{\textbf{w}}$ in $\mathcal {H}^{1+\alpha }$ directly using the Duhamel formulation and exploiting the convergence in Proposition 3.1 and the convergence of $\Pi _{\le N}S(t){\textbf{u}}_0$ and  in $\mathcal {H}^{\alpha }$. $\square$

### Markov semigroups

In this section, we recall how the flows (3.14) and (3.15) generate Markov semigroups on $X^{\alpha }$. We denote by $\mathcal {B}_{b}( X^\alpha )$ the space of measurable, bounded functions from $X^\alpha$ to $\mathbb {R}$, and by $\mathcal {C}_{b}( X^\alpha )$ the space of bounded, continuous functions from $X^\alpha$ to $\mathbb {R}$, both endowed with the norm

$$\begin{aligned} \Vert F\Vert _{L^{\infty }}:=\sup _{{\textbf{u}}_0 \in X^\alpha }|F({\textbf{u}}_0)|. \end{aligned}$$

Given $N\in 2^{\mathbb {N}_0}\cup \{\infty \}$ and $F\in \mathcal {B}_{b}( X^\alpha )$, we define the family of bounded linear operators $\mathcal {P}_{t}^{N}:F\mapsto \mathcal {P}_{t}^NF$, by

3.16 $$\begin{aligned} \mathcal {P}_{t}^NF({\textbf{u}}_0):=\mathbb {E}[ F(\Phi _{t}^N({\textbf{u}}_0;\xi ))], \quad t\ge 0, \end{aligned}$$

for every ${\textbf{u}}_0\in X^\alpha$, and $\mathcal {P}_{t}:=\mathcal {P}_{t}^{\infty }$. We recall from [19, Section 4], that $\mathcal {P}_{t}$ defines a Markov semigroup. The same is true for the operators $\mathcal {P}_{t}^{N}$, $N\in 2^{\mathbb {N}_0}$, defined using the truncated flow. We state the main properties of the semigroups $\{\mathcal {P}_{t}^{N}\}_{N}$. For the proof, see [19, Proposition 4.1, Lemma 4.2, and Proposition 4.3].

#### Lemma 3.3

Let $N\in 2^{\mathbb {N}_0}\cup \{\infty \}$. Then, the following hold true:

(i) Let ${\textbf{u}}_0\in X^\alpha$. Then the map $[0,\infty )\rightarrow X^\alpha$ defined by $t\rightarrow \Phi _{t}^{N}({\textbf{u}}_0;\xi )$ is adapted with respect to the filtration $\{\mathcal {F}_{t}\}_{t\ge 0}$. In particular, for every $t\ge 0$,

3.17 $$\begin{aligned} \Phi ^{N}_{t}({\textbf{u}}_0; \xi ) = \Phi ^{N}_{t}({\textbf{u}}_0 , \textbf{1}_{[0,t]}\xi ) \end{aligned}$$

(ii)For a.e. realisation of $\xi$, and for every $t,h\ge 0$, we have that

3.18 $$\begin{aligned} \Phi ^{N}_{t+h}({\textbf{u}}_0, \xi ) = \Phi ^{N}_{h}(\Phi ^{N}_{t}({\textbf{u}}_0, \textbf{1}_{[0,t]}\xi ), R_{{t}}(\textbf{1}_{[t,\infty )}\xi )), \end{aligned}$$

where $R_{t}$ is the (right) time translation by *t*, which is defined on space-time distributions as an extension of the map $R_{t}f(s,x) = f(s+t,x),$ for $f\in L^1_{\textrm{loc}}(\mathbb {R}\times \mathbb {T}^2)$.

(iii) $\{\Phi _{t}^{N}\}_{t\ge 0}$ is a Markov process and $\{\mathcal {P}_{t}^{N}\}_{t\ge 0}$ is a Markov semigroup.

In fact, more than Lemma 3.3 (iii) is true; namely, the maps $\{\Phi _{t}^{N}\}_{t\ge 0}$ satisfy the strong Markov property (see [19, Section 4]), but we will not need this extra information in this article.

## Modified measures and sample path properties

### Modified measures

The Gaussian measure $\vec {\mu }_s$ in (1.6) can be formally written as

$$\begin{aligned} d\vec {\mu }_{s}= Z_{s}^{-1} e^{-G_{s}(u,v)} du dv, \end{aligned}$$

where

4.1 $$\begin{aligned} G_{s}(u,v) : = \frac{1}{2}\int _{\mathbb {T}^2}(\langle \nabla \rangle ^{s}v)^2 + (\langle \nabla \rangle ^{s+1}u)^2 dx. \end{aligned}$$

Rigorously, we define $\vec {\mu }_s:=\text {Law}({\textbf{u}}^{\omega })= \text {Law}((u^{\omega },v^{\omega }))$, where $(u^{\omega }, v^{\omega })$ denote the following pair of random Fourier series:

$$\begin{aligned} u^{\omega }(x)&= \sum _{n\in \mathbb {Z}^2} \frac{g_{n}(\omega )}{\langle n \rangle ^{s+1}}e^{in\cdot x} \quad \text {and} \quad v^{\omega }(x) = \sum _{n\in \mathbb {Z}^2} \frac{h_{n}(\omega )}{\langle n \rangle ^{s}}e^{in\cdot x}, \end{aligned}$$

where $\{g_n\}_{n\in \mathbb {Z}^2}$ and $\{h_n\}_{n\in \mathbb {Z}^2}$ are families of standard complex-valued Gaussian random variables conditioned so that $g_{n}=\overline{g_{-n}}$ and $h_n=\overline{h_{-n}}$. More precisely, we define the index set

$$\begin{aligned} \Lambda : = (\mathbb {Z}\times \mathbb {Z}_{+})\cup (\mathbb {Z}_{+}\times \{0\}) \cup \{(0,0)\}, \end{aligned}$$

and let $\{g_n,h_n\}_{n\in \Lambda }$ be standard complex valued Gaussian random variables, with $g_0,h_0$ real-valued, and then define $g_{-n}= \overline{g_n}$, $h_{-n}=\overline{h_n}$ for $n\in \mathbb {Z}^2$.

Given $N\in 2^{\mathbb {N}_0}$, we define the marginals

4.2 $$\begin{aligned} \vec {\mu }_{s,N}&:=(\Pi _{\le N})_{*}\vec {\mu }_s =\text {Law}(( \Pi _{\le N}u^{\omega }, \Pi _{\le N}v^{\omega })),\end{aligned}$$

4.3 $$\begin{aligned} \vec {\mu }_{s,N}^{\perp }&:=(\Pi _{> N})_{*}\vec {\mu }_s =\text {Law}(( \Pi _{> N}u^{\omega }, \Pi _{> N}v^{\omega })). \end{aligned}$$

By independence, we then have the decomposition

4.4 $$\begin{aligned} d\vec {\mu }_{s} = d\vec {\mu }_{s,N} \otimes d\vec {\mu }_{s,N}^{\perp }. \end{aligned}$$

We let $\mathcal {V}_{N}$ be the real vector space

$$\begin{aligned} \mathcal {V}_{N} = \text {span}\{ 1, \cos (n\cdot x) , \sin (n\cdot x):\, n\in \Lambda _{N}^{*}\}, \end{aligned}$$

where $\Lambda _{N}^{*}: = \{n\in \mathbb {Z}^2:\, 0<|n|\le N\}\cap \Lambda$, and put the usual scalar product on $\mathcal {V}_{N}$. We endow $\mathcal {V}_N \times \mathcal {V}_N$ with a Lebesgue measure $L_N$ as follows: with

$$\begin{aligned} \Pi _{\le N} u (x) = u_{N}(x) =\sum _{|n|\le N} \widehat{u}_n e^{in\cdot x}, \quad \widehat{u}_{-n} = \overline{\widehat{u}_n}, \end{aligned}$$

we put $a_0=\widehat{u}(0)$, $a_{n,1}= \text {Re}\, \widehat{u}_n$, and $a_{n,2}=\text {Im}\, \widehat{u}_n$ so that

4.5 $$\begin{aligned} u_{N}(x) = a_0 +\sum _{n\in \Lambda _{N}^{*}} \{ a_{n,1}\, 2\cos (n\cdot x) + a_{n,2}(-2\sin (n\cdot x))\}. \end{aligned}$$

We then define $L_N$ as the Lebesgue measure on $\mathcal {V}_N \times \mathcal {V}_N$ with respect to the orthogonal basis

$$\begin{aligned} \big \{ 1, \{ 2 \cos (n\cdot x) , -2\sin (n\cdot x)\}_{n\in \Lambda _{N}^{*}}\big \}\times \big \{ 1, \{ 2 \cos (n\cdot x) , -2\sin (n\cdot x)\}_{n\in \Lambda _{N}^{*}}\big \}. \end{aligned}$$

For every $N\in \mathbb {N}$, the Gaussian measure $\vec {\mu }_{s,N}^{\perp }$ is invariant under the dynamics of the high-frequency equation (3.6). Indeed, a tedious but straightforward computation shows that

$$\begin{aligned} \mathbb {E}[ |\mathcal {F}\{ \pi _1 {\textbf{u}}_{N}^{\perp }\}(t,n)|^{2}] =\langle n \rangle ^{-2s-2} \quad \text {and} \quad \mathbb {E}[ |\mathcal {F}\{ \pi _2 {\textbf{u}}_{N}^{\perp }\}(t,n)|^{2}] =\langle n \rangle ^{-2s} \end{aligned}$$

for all $t\in \mathbb {R}$, and since ${\textbf{u}}_{N}^{\perp }$ evolves linearly, Gaussianity is preserved.

As discussed in [29, 42], it is not clear how to study the quasi-invariance of the measure $\vec {\mu }_{s}$ in (1.6) under the nonlinear flow of (1.1) directly. Indeed, a renormalisation is needed to make sense of the time evolution of $G_{s}$ in (4.1). Thus, following [29], we define

4.6 $$\begin{aligned} \begin{aligned} \sigma _{N}&: = \mathbb {E}_{\vec {\mu }_{s}} \bigg [ \int _{\mathbb {T}^2} (\langle \nabla \rangle ^{s}\Pi _{\le N} u (x))^2 dx \bigg ] \\&= \sum _{|n|_{\infty }, |n|_{\infty } \le N} \frac{\langle n \rangle ^{s}\langle m \rangle ^{s} \mathbb {E}[g_n \overline{g_m}]}{\langle n \rangle ^{s+1}\langle m \rangle ^{s+1}} \\&=\sum _{|n|_{\infty }\le N} \frac{\mathbb {E}[|g_n|^2]}{\langle n \rangle ^{2}} \\&= \sum _{|n|_{\infty }\le N} \frac{1}{\langle n \rangle ^{2}} \sim \log N. \end{aligned} \end{aligned}$$

Then, we set

4.7 $$\begin{aligned} Q_{s,N}(u) : = (\langle \nabla \rangle ^{s} \Pi _{\le N}u)^{2} -\sigma _{N}. \end{aligned}$$

We now define a modified version of (4.1) by

4.8 $$\begin{aligned} E_{s,N}(u,v) = G_{s}(\Pi _{\le N}u, \Pi _{\le N}v) + R_{s,N}(u), \end{aligned}$$

where

4.9 $$\begin{aligned} R_{s,N}(u) : = \frac{3}{2}\int _{\mathbb {T}^2} Q_{s,N}(u)(\Pi _{\le N}u)^{2}dx + \frac{1}{4}\int _{\mathbb {T}^2}(\Pi _{\le N}u)^4 dx. \end{aligned}$$

It follows from Parseval’s theorem that

4.10 $$\begin{aligned} E_{s,N}(u,v) = \mathcal {E}_{s,N}(u, v) + H_{N}(u,v), \end{aligned}$$

where

4.11 $$\begin{aligned} \begin{aligned} \mathcal {E}_{s,N}(u, v)&= \frac{1}{2}\int _{\mathbb {T}^2} (\langle \nabla \rangle m(\nabla ) \Pi _{\le N}u)^2 +(m(\nabla )\Pi _{\le N}v)^{2} + \frac{3}{2}\int _{\mathbb {T}^2} Q_{s,N}(u)(\Pi _{\le N}u)^{2}dx, \end{aligned} \end{aligned}$$

where $m(\nabla ):= \sqrt{\langle \nabla \rangle ^{2s}-1}$, and $H_{N}$ is the energy in (3.13).

The following lemma justifies the construction of the modified measures.

#### Lemma 4.1

Let $s>0$ and $1\le p<\infty$. Then, there exists $R_{s}\in L^{p}(\vec {\mu }_{s})$ such that $R_{s,N}\rightarrow R_{s}$ in $L^{p}(\vec {\mu }_s)$ as $N\rightarrow \infty$. Moreover, there exists $C_{p}>0$ such that

4.12 $$\begin{aligned} \sup _{N\in \mathbb {N}} \big \Vert e^{-R_{s,N}(u)}\big \Vert _{L^{p}(\vec {\mu }_{s})} \le C_p <\infty . \end{aligned}$$

Furthermore,

4.13 $$\begin{aligned} \lim _{N\rightarrow \infty } \big \Vert e^{-R_{s,N}(u,v)} - e^{-R_{s}(u,v)}\big \Vert _{L^{p}(\vec {\mu }_s)}=0, \end{aligned}$$

and thus, for any $\sigma <s$, the restriction to $\mathcal {H}^{\sigma }(\mathbb {T}^2)$ of the measures

4.14 $$\begin{aligned} d\vec {\nu }_{s,N}:=Z_{s,N}^{-1} \, e^{-R_{s,N}(u)} d\vec {\mu }_{s} \end{aligned}$$

converge to a measure

4.15 $$\begin{aligned} d\vec {\nu }_{s}= Z_{s}^{-1} e^{-R_{s}(u)} d\vec {\mu }_{s} \end{aligned}$$

in total variation, where $Z_{s,N}, Z_{s} \in (0,\infty )$ are defined so that $\vec {\nu }_{s,N}$ and $\vec {\nu }_{s}$ are probability measures. In particular, $\vec {\nu }_{s}$ and $\vec {\mu }_s$ are equivalent; that is, $\vec {\nu }_{s} \ll \vec {\mu }_{s}$ and vice versa.

The convergence of $\{R_{s,N}\}_{N\in \mathbb {N}}$ was essentially proved in [42, Lemma 3.4]. The uniform bound (4.12) is possible in view of the positivity of the term $\frac{1}{4}\int u^4 dx$ in (4.9). Our proof of Lemma 4.1 is by now a standard application of the Boué-Dupuis variational formula [1, 5, 55]. However, to our knowledge a proof for the whole range $s>0$ does not appear in the literature, so for the reader’s convenience, we will present some of the details in Section 4.3.

Note that

4.16 $$\begin{aligned} Z_{s,N}:=\int e^{-R_{s,N}(u)}d\vec {\mu }_{s}(u) = \int e^{-R_{s,N}(u_N)}d\mu _{s,N}(u_N). \end{aligned}$$

In particular, by Jensen’s inequality and Lemma 4.1, there is a $C>0$ such that

4.17 $$\begin{aligned} \inf _{N\in \mathbb {N}} Z_{s,N} \ge C>0. \end{aligned}$$

#### Remark 4.2

The choice of the energy functional (4.8) is identical to the choice made in the deterministic setting [42]. While this choice can be justified a posteriori due to the arguments in Section 5, it should be expected that the two choices should correspond, and we now try to explain this via a finite-dimensional analogy. Consider a second-order system of ODEs on $\mathbb {R}^d$ of the form

4.18 $$\begin{aligned} \frac{d^2}{dt^2} u = b(u), \end{aligned}$$

and denote its flow by $\Psi _t({\textbf{u}}_0)$, and the system of SDEs

4.19 $$\begin{aligned} \frac{d^2}{dt^2} u + \frac{d}{dt} u = b(u) + \dot{W}, \end{aligned}$$

where *W* denotes a standard d-dimensional Brownian motion, and denote by $P_t$ the semigroup generated by this system. Suppose moreover (for convenience) that *b* is divergence-free. Let

$$\begin{aligned} \vec {\rho }_0(u,u_t) = \rho _0(u) \exp \left( -\frac{1}{2} |u_t|^2\right) du du_t \end{aligned}$$

be a measure on $\mathbb {R}^d \times \mathbb {R}^d$, and for convenience denote $\mu (u_t) = \exp \left( -\frac{1}{2} |u_t|^2\right)$. Then, by Liouville theorem and the Kolmogorov/Fokker-Plank equations respectively, we have that

$$\begin{aligned} \partial _t \big ((\Psi _t)_*\vec {\rho }_0\big )\Big |_{t = 0} = - \big (u_t \cdot \nabla \rho _0(u)\big ) \mu (u_t) - \big (b(u) \cdot \nabla \mu _0(u_t)\big ) \rho _0(u) =: \mathcal Q(u,u_t) \vec {\rho }_0 (u,u_t), \end{aligned}$$

and

$$\begin{aligned} \partial _t \big (P_t^* \vec {\rho }_0\big )\Big |_{t = 0}&= \mathcal Q(u,u_t) \vec {\rho }_0 (u,u_t) +\Big ( - \textrm{div}\big (u_t \mu (u_t)\big ) + \frac{1}{2} (\Delta \mu )(u_t)\Big )\rho _0(u) \\&= \mathcal Q(u,u_t) \vec {\rho }_0 (u,u_t), \end{aligned}$$

namely, the time derivatives in 0 are the same. This is essentially due to the fact that the “extra" terms of (4.19) when compared to (4.18) leave the Gaussian measure $\mu$ invariant. By another computation, it is straightforward to check that the quantities appearing within the exponential in (1.9) and (1.10) are exactly

$$\begin{aligned} \partial _t \big ((\Psi _t)_*\vec {\rho }_0\big )\Big |_{t = 0}, \quad \partial _t \big (P_t^* \vec {\rho }_0\big )\Big |_{t = 0} \end{aligned}$$

respectively, which explains why a good estimate for one should translate into a good estimate for the other.

### Pathwise properties

The goal of this section is to establish some properties that sample paths of $\vec {\nu }_s$ and $\xi$ satisfy, building up to the uniform bounds on the truncated flows $\Phi ^{N}_{t}$ in Lemma 4.5 and Lemma 4.6. We begin with samples of $\vec {\mu }_{s}$, which enjoy the following regularity property.

#### Lemma 4.3

Let $\alpha <s$. For ${\textbf{u}}\sim \vec {\mu }_{s}$, we have ${\textbf{u}}\in Z^{\alpha }\subset \mathcal {C}^{\alpha }$ almost surely. Moreover,

4.20 $$\begin{aligned} \mathbb {E}\bigg [ \sup _{N\in 2^{\mathbb {N}_0} \cup \{\infty \}}\Vert \Pi _{\le N} \Phi _{t}^{lin }({\textbf{u}},\xi )\Vert _{C([0,T];\mathcal {C}^{\alpha })}^{p} \bigg ] \le C(p,T). \end{aligned}$$

#### Proof

Clearly, the membership in $Z^{\alpha }$ is stronger than that in $\mathcal {C}^{\alpha }$, so we focus on the former. Using Sobolev embedding for some $p<\infty$ large enough so that $\frac{2}{p}+\alpha <s$, with $q\ge p$, Lemma 2.8, we have

$$\begin{aligned} \mathbb {E}[ \Vert {\textbf{u}}\Vert _{Z^{\alpha }}^{q}]&\lesssim \sum _{j=0}^{\infty } e^{\frac{j}{8}} \mathbb {E}\Big [ \sup _{t\in [j,j+1]} \Vert S(t){\textbf{u}}\Vert _{\mathcal {W}^{\alpha +\frac{2}{p},p}}^{q} \Big ] \\&\lesssim \sum _{j=0}^{\infty } e^{\frac{j}{8}} \bigg \{ \mathbb {E}\Big [ \Vert S(j){\textbf{u}}\Vert _{\mathcal {W}^{\alpha +\frac{2}{p},p}}^{q}\Big ] + \sup _{j\le t' <t\le j+1} \mathbb {E}\bigg [\frac{ \Vert S(t){\textbf{u}}- S(t'){\textbf{u}}\Vert _{\mathcal {W}^{\alpha +\frac{2}{p},p}}^{q}}{|t-t'|^{1+\gamma q +\varepsilon }} \bigg ] \bigg \}, \end{aligned}$$

for some $\gamma >0$ to chosen later. It will suffice to control the norms of just the first components of the vectors. For each fixed $x\in \mathbb {T}^2$, $\langle \nabla \rangle ^{\alpha +\frac{2}{p}} \pi _1 S(j){\textbf{u}}(x)$ is a mean zero Gaussian random variable with a variance bounded by

4.21 $$\begin{aligned} \text {Var}(\langle \nabla \rangle ^{\alpha +\frac{2}{p}} \pi _1 S(j){\textbf{u}}(x))\lesssim e^{-j} \sum _{n} \frac{\langle n \rangle ^{2\alpha +\frac{4}{p}}}{\langle n \rangle ^{2+2s}} \lesssim e^{-j}. \end{aligned}$$

By Minkowski’s inequality, the Gaussianity of $\langle \nabla \rangle ^{\alpha +\frac{2}{p}} \pi _1 S(j){\textbf{u}}(x)$ and (4.21),

$$\begin{aligned} \mathbb {E}[ \Vert \pi _1 S(j){\textbf{u}}\Vert _{W^{\alpha +\frac{2}{p},p}}^{q}]^{\frac{1}{q}} \le \bigg \Vert \mathbb {E}[ |\langle \nabla \rangle ^{\alpha +\frac{2}{p}} \pi _1 S(j){\textbf{u}}(x)|^{q}]^{\frac{1}{q}} \bigg \Vert _{L^{p}_{x}} \lesssim e^{-\frac{j}{2}}q^{\frac{1}{2}}. \end{aligned}$$

The factor $e^{-j/2}$ ensures the summability of the first term in *j*. For the second term we argue similarly using the mean value theorem to see that for each fixed $x\in \mathbb {T}^2$, $\langle \nabla \rangle ^{\alpha +\frac{2}{p}} \pi _1 S(t){\textbf{u}}(x)-\langle \nabla \rangle ^{\alpha +\frac{2}{p}} \pi _1 S(t'){\textbf{u}}(x)$ is a mean zero Gaussian random variable with variance bounded by

$$\begin{aligned} e^{-j} \sum _{n\in \mathbb {Z}} \frac{\langle n \rangle ^{2\theta +2\alpha +\frac{4}{p}}|t-t'|^{2\theta }}{ \langle n \rangle ^{2+2s} } \lesssim e^{-j} |t-t'|^{2\theta }, \end{aligned}$$

provided that $0<\theta < \min (1, s-\alpha -\frac{2}{p})$. Thus,

$$\begin{aligned} \sup _{j\le t' <t\le j+1} \mathbb {E}\bigg [\frac{ \Vert S(t){\textbf{u}}- S(t'){\textbf{u}}\Vert _{\mathcal {W}^{\alpha +\frac{2}{p},p}}^{q}}{|t-t'|^{1+\gamma q +\varepsilon }} \bigg ] \lesssim e^{-q\frac{j}{2}} q^{\frac{q}{2}}, \end{aligned}$$

provided that $\gamma <\theta -\frac{1}{q}-\frac{\varepsilon }{q}$ which is possible by taking *q* sufficiently large and $\varepsilon$ sufficiently small at the beginning. We then obtain

$$\begin{aligned} \mathbb {E}[ \Vert {\textbf{u}}\Vert _{Z^{\alpha }}^{q}] \lesssim q^{\frac{1}{2}}. \end{aligned}$$

As for (4.20), this follows in a similar way as for (2.40). In particular, by (3.8) and the triangle inequality, we only need to prove the bound for $S(t){\textbf{u}}$, which further reduces to showing

4.22 $$\begin{aligned} \mathbb {E}\big [ \Vert \Pi _{\le 1}S(t){\textbf{u}}\Vert _{C([0,T];\mathcal {C}^{\alpha })}^{p}\big ]&\lesssim _{p,T} 1,\end{aligned}$$

4.23 $$\begin{aligned} \mathbb {E}\big [ \Vert \Pi _{\le 2M}S(t){\textbf{u}}-\Pi _{\le M}S(t){\textbf{u}}\Vert _{C([0,T];\mathcal {C}^{\alpha })}^{p}]&\lesssim _{p,T} M^{-\theta } \end{aligned}$$

for some $\theta >0$, and every $M\in 2^{\mathbb {N}_0}$. Here, (4.22) just follows from Sobolev embedding and (2.16). Now, (4.23) follows similar arguments as above in showing ${\textbf{u}}\in Z^{\alpha }$, and we gain the factor $M^{-\theta }$ from localisation to frequencies around *M* in the sum (4.21). $\square$

As our arguments in Section 5.3 require us to also control norms of the time evolution of the function $Q_{s,N}(u)$, we need to be more precise here about this quantity. For $u\in C^{\alpha }(\mathbb {T}^2)$, we define

4.24 $$\begin{aligned} Q_{s}(u) := \lim _{N\rightarrow \infty } Q_{s,N}(u) \end{aligned}$$

in $H^{\alpha -s}(\mathbb {T}^2)$, whenever this limit exists.

#### Lemma 4.4

Let $\alpha <s$ but sufficiently close to *s*. Suppose that $u\in C^{\alpha }(\mathbb {T}^2)$ is such that $Q_{s}(u)$ exists in the sense of (4.24), and let $w\in H^{1+\alpha }(\mathbb {T}^2)$. Then, $Q_{s}(u+w)$ exists in the sense of (4.24) and satisfies

4.25 $$\begin{aligned} Q_{s}(u+w) = Q_{s}(u) + 2(\langle \nabla \rangle ^s u)(\langle \nabla \rangle ^s w)+(\langle \nabla \rangle ^s w)^2. \end{aligned}$$

In particular, if *u* is distributed according to $\mu _{s+1}$, then almost surely $Q_{s}(u)$ exists, and moreover, $Q_{s}(u)\in C^{-\varepsilon }(\mathbb {T}^2)$ for any $\varepsilon >s-\alpha$, and $Q_{s,N}(u)\rightarrow Q_{s}(u)$ in $C^{-\varepsilon }(\mathbb {T}^2)$. Finally, for ${\textbf{u}}_0$ distributed according to $\vec {\mu }_{s}$, $Q_{s}(\pi _1 \Phi _{t}^{lin }({\textbf{u}}_0,\xi ))$ exists almost surely and satisfies

4.26 $$\begin{aligned} \mathbb {E}\bigg [ \sup _{N\in 2^{\mathbb {N}_0}\cup \{\infty \}} \Vert Q_{s,N}(\pi _{1}\Phi _{t}^{lin }({\textbf{u}}_0,\xi ))\Vert _{C([0,T];H^{-\sigma })}^p\bigg ] \le C(T,p). \end{aligned}$$

for any $1\le p<\infty$, $\sigma >0$, and where $\Phi _{t}^{lin }$ is the linear flow defined in (3.7).

#### Proof

For each fixed $N\in \mathbb {N}$, it follows by the definition (4.7) that

4.27 $$\begin{aligned} Q_{s,N}(u+w) = Q_{s,N}(u) + 2(\langle \nabla \rangle ^{s}\Pi _{\le N}u)(\langle \nabla \rangle ^s \Pi _{\le N}w)+ (\langle \nabla \rangle ^s \Pi _{\le N}w)^2. \end{aligned}$$

Then, the existence of $Q_{s}(u+w)$ is ensured by verifying that the right hand side of (4.27) converges as $N\rightarrow \infty$. The term $Q_{s,N}(u)$ converges by our assumption on *u*. For the mixed term $(\langle \nabla \rangle ^{s}\Pi _{\le N}u)(\langle \nabla \rangle ^s \Pi _{\le N}w)$, we use (2.13) which is controlled provided that $1+\alpha -s>s-\alpha$, which holds as long as $\alpha <s$ is sufficiently close to *s*. Similarly, $(\langle \nabla \rangle ^{s} \Pi _{\le N}w)^{2}$ converges to $(\langle \nabla \rangle ^{s} w)^{2}$ in $H^{\alpha -s}$, as long as $1+\alpha -s>s-\alpha$.

The second claim regarding functions *u* distributed according to $\mu _{s+1}$ is essentially shown in [29, (4.6), Proposition 4.3] and the required details are already encapsulated in showing (4.26), which we move onto now.

By a telescoping series argument, it is enough to show that

4.28 $$\begin{aligned} \mathbb {E}\big [ \Vert Q_{s,1}(\pi _1 \Phi ^{\text {lin}}_{t}({\textbf{u}}_0,\xi ))\Vert _{C([0,T];H^{-\sigma })}^{p}\big ]&\lesssim _{p,T} 1,\end{aligned}$$

4.29 $$\begin{aligned} \mathbb {E}\big [ \Vert Q_{s,2M}(\pi _1 \Phi ^{\text {lin}}_{t}({\textbf{u}}_0,\xi ))-Q_{s,M}(\pi _1 \Phi ^{\text {lin}}_{t}({\textbf{u}}_0,\xi ))\Vert _{C([0,T];H^{-\sigma })}^{p}]&\lesssim _{p,T} M^{-\delta } \end{aligned}$$

for $1\le p<\infty$ and some $\delta >0$. In particular, (4.29) also establishes that the sequence $\{Q_{s,N}(\pi _1\Phi _{t}^{\text {lin}}({\textbf{u}}_0,\xi ))\}_{N}$ is Cauchy in this sense and thus $Q_{s}(\pi _{1} \Phi _{t}^{\text {lin}}({\textbf{u}}_0,\xi ))$ exists in $L^p( \Omega ; C([0,T];H^{-\sigma }))$. We only consider (4.29) as simpler arguments suffice for (4.28).

By Lemma 2.8, we have

4.30 $$\begin{aligned} \begin{aligned}&\mathbb {E}\big [ \Vert Q_{s,2M}(\pi _1 \Phi ^{\text {lin}}_{t}({\textbf{u}}_0,\xi ))-Q_{s,M}(\pi _1 \Phi ^{\text {lin}}_{t}({\textbf{u}}_0,\xi ))\Vert _{C([j,j+1];H^{-\sigma })}^{p}] \\&\lesssim \mathbb {E}\big [ \Vert \mathcal {Q}_{s,M}(t)\Vert _{H^{-\sigma }}^{p}] +\sup _{j\le t'<t\le j+1} \mathbb {E}\bigg [ \frac{ \Vert \mathcal {Q}_{s,M}(t)-\mathcal {Q}_{s,M}(t') \Vert _{H^{-\sigma }}^{p} }{|t-t'|^{1+p\gamma +\varepsilon }} \bigg ] , \end{aligned} \end{aligned}$$

where $\gamma <\theta -\frac{1+\varepsilon }{p}$, for some $0<\theta <1$, and

$$\begin{aligned} \mathcal {Q}_{s,M}(t): =Q_{s,2M}(\pi _1 \Phi ^{\text {lin}}_{t}({\textbf{u}}_0,\xi ))-Q_{s,M}(\pi _1 \Phi ^{\text {lin}}_{t}({\textbf{u}}_0,\xi )). \end{aligned}$$

For each fixed $t\ge 0$, the law of $\Phi _{t}^{\text {lin}}({\textbf{u}}_0,\xi )$ is $\vec {\mu }_{s}$, so then

$$\begin{aligned} \mathbb {E}\Big [ \Vert \mathcal {Q}_{s,M}(j)\Vert _{H^{-\sigma }}^{2} \Big ]&= \mathbb {E}\Big [ \Vert \mathcal {Q}_{s,M}(0)\Vert _{H^{-\sigma }}^{2} \Big ]\\&\lesssim \sum _{n\ne 0} \frac{1}{\langle n \rangle ^{2\sigma }} \sum _{ \begin{array}{c} n=n_{1}+n_2\\ \exists j\in \{1,2\}, |n_j|\sim M \end{array}} \prod _{j=1}^{2} \frac{1 }{\langle n_1 \rangle ^2 \langle n_2 \rangle ^{2}} + \sum _{M<|n|\le 2M} \frac{1}{\langle n \rangle ^{4}} \\&\lesssim (\log M )M^{-2\sigma } +M^{-2}, \end{aligned}$$

which is acceptable as $\sigma >0$. The Wiener Chaos estimate (see [47, Theorem I.22] and [50, Proposition 2.4]) then implies a similar result for any finite $p>2$. Moreover, by the Wiener Chaos estimate, for the second term in (4.30), it is enough to show that

4.31 $$\begin{aligned} \mathbb {E}\Big [ \Vert \mathcal {Q}_{s,M}(t)-\mathcal {Q}_{s,M}(t')\Vert _{H^{-\sigma }}^{2} \Big ] \lesssim |t-t'|^{2\theta }, \end{aligned}$$

where $\gamma <\theta -\frac{1+\varepsilon }{p}$ and for some $0<\theta <1$. By expanding the definition of the $H^{-\sigma }$ norm, (4.31) follows from

4.32 $$\begin{aligned} \mathbb {E}\Big [ \big | \mathcal {F}\{\mathcal {Q}_{s,M}(t)-\mathcal {Q}_{s,M}(t')\}(n) \big |^{2} \Big ] \lesssim |t-t'|^{2\theta } \langle n \rangle ^{-2+2\theta +2\delta }M^{-\delta }, \end{aligned}$$

for any $n\in \mathbb {Z}$. We compute

4.33 $$\begin{aligned} \begin{aligned} \mathcal {F}\{\mathcal {Q}_{s,M}(t)\}(n)&= \sum _{ \begin{array}{c} n=n_{1}+n_2\\ n\ne 0 \\ \exists j\in \{1,2\}, |n_j|\sim M \end{array}}\prod _{j=1}^{2} \frac{z(t,n_j)}{\langle n_j \rangle } + \textbf{1}_{\{n=0\}} \sum _{M<|m|\le 2M} \frac{|z(t,m)|^2-1}{\langle m \rangle ^2} \end{aligned} \end{aligned}$$

where

$$\begin{aligned} z(t,n) : = e^{-\frac{t}{2}} \bigg ( ( \cos (t[ n ])+\tfrac{\sin (t[ n ])}{2[ n ]} )g_n&+ \tfrac{\langle n \rangle }{ [ n ]}\sin (t[ n ])h_n \\&+ \tfrac{\sqrt{2}\langle n \rangle }{[ n ]} \int _{0}^{t} e^{\frac{t'}{2}}\sin ((t-t')[ n ])d\beta _{n}(t') \bigg ). \end{aligned}$$

Then, taking the difference of the first term in (4.33) at times $j\le t'<t\le j+1$, with the absolute value squared, and using the mean value theorem, we find

4.34 $$\begin{aligned} \begin{aligned} \sum _{\begin{array}{c} n=n_1+n_2 \\ \max (|n_1|,|n_2|)\sim M \end{array} } \frac{ \max (\langle n_1 \rangle ,\langle n_2 \rangle )^{2\theta }}{\langle n_1 \rangle ^2\langle n_2 \rangle ^2} |t-t'|^{2\theta }&\lesssim |t-t'|^{2\theta } \langle n \rangle ^{-2+2\theta +2\delta }M^{-\delta }, \end{aligned} \end{aligned}$$

provided that $0<\theta <1$. When $n=0$, we need to use that fact that for each fixed $t\ge 0$ and $m\in \mathbb {Z}$, *z*(*t*, *m*) is a standard complex Gaussian random variable so

$$\begin{aligned} |z(t,m)|^2 -1 = |z(t,m)|^2- \mathbb {E}[|z(t,m)|^2]. \end{aligned}$$

Then, (4.32) follows in this case since

$$\begin{aligned} \mathbb {E}[ (|z(t,m_1)|^2-|z(t',m_1)|^2)&(|z(t,m_2)|^2-|z(t',m_2)|^2)] \\&= \delta _{m_1,m_2} \mathbb {E}[ (|z(t,m_1)|^2-|z(t',m_1)|^2)^2], \end{aligned}$$

and thus

$$\begin{aligned} \mathbb {E}\Big [ \big | \mathcal {F}\{\mathcal {Q}_{s,M}(t)-\mathcal {Q}_{s,M}(t')\}(0) \big |^{2} \Big ] \lesssim |t-t'|^{2\theta } \sum _{|m|\sim M} \frac{1}{\langle m \rangle ^{4-2\theta }} \lesssim |t-t'|^{2\theta } M^{-2+2\theta } \end{aligned}$$

for any $0<\theta <1$. This completes the proof of (4.32) and thus also (4.31). $\square$

We now show that (4.20) and (4.26) also hold for the nonlinear flow $\Phi _{t}$ of (1.1). To this end, we need to exploit that ${\textbf{w}}$ belongs to the higher regularity space $\mathcal {H}^{1+s-\varepsilon }$ for any $\varepsilon >0$, namely, one full degree better than that of the initial data ${\textbf{u}}_0$ and the stochastic convolution . It is convenient to verify this a posteriori relative to the results from Proposition 3.1. In particular, the following results hold pathwise.

#### Lemma 4.5

Let $0<\alpha '<\alpha <s$, $T>0$, and ${\textbf{u}}_0 \in X^{\alpha }$. Then, for every $N\in 2^{\mathbb {N}_0}\cup \{\infty \}$, ${\textbf{w}}_{N}(t)\in C([0,T];\mathcal {H}^{1+\alpha }(\mathbb {T}^2))$, and whenever4.35for some $R>0$, there exists $C(T,R)>0$ such that

4.36 $$\begin{aligned} \sup _{N,M\in 2^{\mathbb {N}_0}\cup \{\infty \}}\sup _{t\in [0,T]} \Big \{ \Vert \Pi _{\le M} {\textbf{w}}_{N}(t)\Vert _{\mathcal {C}^{\alpha '}}+ \Vert {\textbf{w}}_{N}(t)\Vert _{\mathcal {H}^{1+\alpha }} \Big \} \le C(T,R). \end{aligned}$$

#### Proof

By (3.11), (2.16), the fractional Leibniz rule, and the definitions of the space $X^{\alpha }$, we get4.37Now, the bound in $\mathcal {H}^{1+\alpha }$ in (4.36) follows from (4.35) and (3.12). For the $\mathcal {C}^{\alpha '}$-norm in (4.36), we use the embedding $\mathcal {H}^{1+\alpha '+\varepsilon }\hookrightarrow \mathcal {C}^{\alpha '}$ for any $\varepsilon >0$, the boundedness of $\Pi _{\le M}$ on $\mathcal {H}^{1+\alpha }$, and the bound in $\mathcal {H}^{1+\alpha }$ that we just proved. $\square$

We now obtain a uniform control on the transport of the renormalised quantities $Q_{s,M}$.

#### Lemma 4.6

Let $0<\alpha <s$ such that $s-\alpha < \frac{1}{2}$. Given $T>0$ and $R> 0$, suppose thatThen, there exists a constant $C(T,R)>0$ such that

4.38 $$\begin{aligned} \sup _{N,M\in 2^{\mathbb {N}_0}}\sup _{t\in [0,T]} \Vert Q_{s,M}(\pi _1 \Phi _{t}^{N}({\textbf{u}}_0,\xi ))\Vert _{H^{\alpha -s}} \le C(T,R). \end{aligned}$$

#### Proof

By Sobolev embedding, our assumption, and (4.36),

$$\begin{aligned} \Vert (\langle \nabla \rangle ^s \pi _1 \Pi _{\le M} {\textbf{w}}_{N}(t))^{2}\Vert _{H^{\alpha -s}}&\le \Vert \pi _1 \Pi _{\le M}{\textbf{w}}_{N}(t)\Vert _{W^{s,4}}^{2} \lesssim \Vert \pi _1 {\textbf{w}}_{N}(t)\Vert _{H^{s+\frac{1}{2}}}^{2} \le C(R,T), \end{aligned}$$

uniformly in *M* and *N*, and where we used that $s+\frac{1}{2}\le 1+\alpha$, when $s-\alpha \le \frac{1}{2}$. Next, using (2.13) and (4.36), we get

$$\begin{aligned} \Vert (\langle \nabla \rangle ^s \pi _1&\Pi _{\le M}\Phi _{t}^{\text {lin}}({\textbf{u}}_0,\xi ))( \langle \nabla \rangle ^{s}\pi _{1} \Pi _{\le M}{\textbf{w}}_N (t)) \Vert _{H^{\alpha -s}} \\&\lesssim \Vert \langle \nabla \rangle ^s \pi _1 \Pi _{\le M}\Phi _{t}^{\text {lin}}({\textbf{u}}_0,\xi )\Vert _{C^{\alpha -s}} \Vert \langle \nabla \rangle ^{s}\pi _{1} {\textbf{w}}_N (t)\Vert _{H^{\alpha -s+\varepsilon }} \\&\lesssim \Vert \pi _1 \Pi _{\le M}\Phi _{t}^{\text {lin}}({\textbf{u}}_0,\xi )\Vert _{C^{\alpha }} \Vert \pi _{1} {\textbf{w}}_{N}(t)\Vert _{H^{1+\alpha }} \\&\le C(R,T), \end{aligned}$$

provided that $\varepsilon <1$. Combining these estimates, recalling (3.14) and (3.7) and then applying (4.25) and the triangle inequality yields (4.38). $\square$

### Proof of Lemma 4.1

First, we note that (4.13) follows from (4.12) and the convergence in measure from of (4.9), which in turn follows from immediately from Lemma 4.4. We thus focus on establishing (4.12). We set $p=1$ as the argument is identical for any finite $p>1$. We now state the Boué-Dupuis variational formula [5, 55]; the current version can be found in [20, Lemma 2.6].

#### Lemma 4.7

Let *Y* be distributed according to $(\pi _{1})_{*}\vec {\mu }_{s}$ and $N \in \mathbb {N}$. Suppose that $F:C^\infty (\mathbb {T}^2) \rightarrow \mathbb {R}$ is measurable such that $\mathbb {E}\big [|F_{-}(\Pi _{\le N}Y)|^p\big ] < \infty$ for some $p>1$, where $F_{-}$ denotes the negative part of *F*. Then, we have

4.39 $$\begin{aligned} \log \mathbb {E}\Big [e^{-F(\Pi _{\le N} Y)}\Big ] \le \mathbb {E}\bigg [ \sup _{\Theta \in H^{1+s}}\Big \{ -F(\Pi _{\le N} Y + \Pi _{\le N} \Theta ) - \tfrac{1}{2} \Vert \Theta \Vert _{H^{1+s}_x}^2 \Big \} \bigg ], \end{aligned}$$

and the expectation $\mathbb {E}= \mathbb {E}_{\mathbb {P}}$ is an expectation with respect to the underlying probability measure $\mathbb {P}$.

In the following, we simply write $Y_{N}$ for $\Pi _{N}Y$ and $\Theta _{N}$ for $\Pi _{\le N}\Theta$.

#### Lemma 4.8

Let $s>0$. Then, given any finite $p \ge 1$, we have

$$\begin{aligned} \begin{aligned} \mathbb {E}\Big [&\Vert Y_N\Vert _{C^{s-\varepsilon }}^p + \Vert Q_{s,N}(Y_N)\Vert _{C^{-\varepsilon }}^p \Big ] \le C_{\varepsilon , p} <\infty , \end{aligned} \end{aligned}$$

for any $\varepsilon >0$, and uniformly in $N \in \mathbb {N}$

We omit the proof of Lemma 4.8 as it follows similarly from [29, (4.6), Proposition 4.3].

#### Proof of Lemma 4.1

In view of (4.16), we apply Lemma 4.7 with $F=R_{s,N}$. Note that the assumptions in Lemma 4.7 are trivially satisfied as the bounds may depend on *N*. From (4.39), we have

4.40 $$\begin{aligned} \begin{aligned} \log Z_{s,N}&\le \mathbb {E}\Big [ \sup _{\Theta \in H^{1+s}}\Big \{ -R_{s,N}(Y_N +\Theta _N) -\tfrac{1}{2}\Vert \Theta _N\Vert _{H^{1+s}}^{2} \Big \}\Big ]. \end{aligned} \end{aligned}$$

By Hölder and Young’s inequalities, we have

4.41 $$\begin{aligned} \bigg | \int _{\mathbb {T}^2} Y_{N}^{3} \Theta _{N} dx \bigg | +\bigg | \int _{\mathbb {T}^2} Y_{N} \Theta _{N}^3 dx \bigg |&\le C\Vert Y_N\Vert _{L^{4}}^{4} +\frac{1}{100}\Vert \Theta _{N}\Vert _{L^{4}}^{4}. \end{aligned}$$

Thus, (4.41) implies

4.42 $$\begin{aligned} -\frac{1}{8}\int _{\mathbb {T}^2}(Y_N+\Theta _N)^{4}dx \le -\frac{1}{16}\Vert \Theta _N\Vert _{L^{4}}^{4} + C\Vert Y_N\Vert _{L^{4}}^{4}. \end{aligned}$$

Next, by (2.5), (2.6), (2.3), for every $\delta >0$, there exists $C_{\delta }>0$ such that

4.43 $$\begin{aligned} \begin{aligned} \bigg | \int _{\mathbb {T}^2} Q_{s,N}(Y_N)(Y_N +\Theta _N)^2 dx \bigg |&\le \Vert Q_{s,N}(Y_N)\Vert _{C^{-\frac{\varepsilon }{2}}} \Vert (Y_N+\Theta _N)^{2}\Vert _{B^{\frac{\varepsilon }{2}}_{1,1}} \\&\lesssim \Vert Q_{s,N}(Y_N)\Vert _{C^{-\frac{\varepsilon }{2}}}\Vert Y_N+\Theta _N\Vert _{L^2} \Vert Y_N+\Theta _N\Vert _{B^{\frac{\varepsilon }{2}}_{2,1}} \\&\lesssim \Vert Q_{s,N}(Y_N)\Vert _{C^{-\frac{\varepsilon }{2}}}\Vert Y_N+\Theta _N\Vert _{L^2} \Vert Y_N +\Theta _N\Vert _{H^{\varepsilon }} \\&\le C_{\delta } (1+\Vert Q_{s,N}(Y_N)\Vert _{C^{-\frac{\varepsilon }{2}}})^{2}(1+\Vert Y_N\Vert _{H^{\varepsilon }})^{4} \\&+ \delta ( \Vert \Theta _N\Vert _{L^{4}}^{4} +\Vert \Theta _N\Vert _{H^{1+s}}^{2}). \end{aligned} \end{aligned}$$

By Hölder and Young’s inequalities, we also have

4.44 $$\begin{aligned} \bigg | \int _{\mathbb {T}^2} Y_N \Theta _N (Y_N +\Theta _N)^2 dx \bigg |&\le C_{\delta }(1+\Vert Y_N\Vert _{L^{\infty }})^{4} + \delta \Vert \Theta _N\Vert _{L^{4}}^{4}, \end{aligned}$$

for any $\delta >0$. Thus, by (4.43) and (4.44), we have

4.45 $$\begin{aligned} \begin{aligned}&-\int _{\mathbb {T}^2} Q_{s,N}(Y_N+\Theta _N)(Y_N +\Theta _N)^2 dx \\&\le - \int _{\mathbb {T}^2} Q_{s,N}(Y_N)(Y_N +\Theta _N)^2 dx -2\int _{\mathbb {T}^2} Y_N \Theta _N (Y_N +\Theta _N)^2 dx \\&\le C( \Vert Q_{s,N}(Y_N)\Vert _{C^{-\frac{\varepsilon }{2}}}, \Vert Y_N\Vert _{H^{\varepsilon }}, \Vert Y_{N}\Vert _{L^{\infty }}) + \delta ( \Vert \Theta _N\Vert _{L^{4}} + \Vert \Theta _{N}\Vert _{H^{1+s}}^{2}), \end{aligned} \end{aligned}$$

where *C*(*x*, *y*, *z*) is polynomial in its arguments. Therefore Lemma 4.8 implies

$$\begin{aligned} \sup _{N\in \mathbb {N}}\mathbb {E}[ C( \Vert Q_{s,N}(Y_N)\Vert _{C^{-\frac{\varepsilon }{2}}}, \Vert Y_N\Vert _{H^{\varepsilon }}, \Vert Y_{N}\Vert _{L^{\infty }}) ] <\infty . \end{aligned}$$

Combining (4.42) and (4.45) in (4.40) and choosing $\delta >0$ sufficiently small, we have shown

$$\begin{aligned} \log Z_{s,N} \le C+ \mathbb {E}\big [ \sup _{\Theta \in H^{1+s}}\big \{ -\tfrac{1}{32}\Vert \Theta _N\Vert _{L^{4}}^{4} -\tfrac{1}{4}\Vert \Theta _N\Vert _{H^{1+s}}^{2} \big \} \big ] \le C, \end{aligned}$$

uniformly in $N\in \mathbb {N}$. This completes the proof of (4.12). $\square$

## On the transported densities

### Functional derivatives

For vector-valued functions ${\textbf{u}}$ and ${\textbf{v}}$, we define an inner product on $L^2(\mathbb {T}^2)\times L^2(\mathbb {T}^2)$ by

$$\begin{aligned} \langle {\textbf{u}}, {\textbf{v}} \rangle _{L^2\times L^2} : = \langle u_1, v_1 \rangle _{L^2} + \langle u_2,v_2 \rangle _{L^2}, \end{aligned}$$

and we denote the Fréchet derivatives as follows: if $F:\mathcal {S}(\mathbb {R})^2 \rightarrow \mathbb {R}$, then

$$\begin{aligned} dF\vert _{{\textbf{u}}}[ \textbf{f}] = \frac{d}{d\theta }\bigg \vert _{\theta =0} F({\textbf{u}}+\theta \textbf{f}) =\langle D_{{\textbf{u}}}F, \textbf{f} \rangle = \langle \partial _{u_1}F, f_1 \rangle +\langle \partial _{u_2}F, f_2 \rangle . \end{aligned}$$

and, if *F* is also $C^2$, then we define the second functional derivative

$$\begin{aligned} D^{2}F\vert _{{\textbf{u}}}[\textbf{f}, \textbf{h}] := \frac{d}{d\theta }\bigg \vert _{\theta =0} dF|_{{\textbf{u}}+\theta \textbf{h}} [ \textbf{f}] = \langle (D^{2}_{{\textbf{u}}} F)[ \textbf{h}], \textbf{f} \rangle \end{aligned}$$

and the Hessian $D^{2}_{{\textbf{u}}}F$ can be further expanded as

$$\begin{aligned} D^{2}_{{\textbf{u}}}F = \begin{pmatrix} \partial _{u_1 u_2}^{2} F &  \partial _{u_1 u_2}^{2}F \\ \partial _{u_2 u_1}^{2}F &  \partial _{u_2 u_2}^{2}F \end{pmatrix}. \end{aligned}$$

In the finite dimensional setting, we can relate the functional derivatives $D_{{\textbf{u}}_N}$ to the derivatives with respect to the Fourier coefficients $\{a_0, a_{n,1}, a_{n,2}\}_{n\in \Lambda _{N}^{*}}$ of $u_N$ as in (4.5). In particular, by the chain rule, we have

$$\begin{aligned} \partial _{a_{n,1}}F = \Re \widehat{\partial _{u_N}F}(n), \quad \partial _{a_{n,2}}F = \Im \widehat{\partial _{u_N}F}(n), \quad \text {and} \quad \partial _{a_0}F = \widehat{\partial _{u_N}F}(0), \end{aligned}$$

so that

$$\begin{aligned} \partial _{u_N}F(x) = \partial _{a_{0}}F + \sum _{n\in \Lambda _{N}^{*}} \big \{ \partial _{a_{n,1}}F \cdot 2\cos (n\cdot x) + \partial _{a_{n,2}}F \cdot (-2\sin (n\cdot x))\big \}. \end{aligned}$$

### Transported densities

In the following, we fix $N\in 2^{\mathbb {N}_0}$ and consider the truncated system (3.5). Using $\Pi _{\le N} \xi (t) = \sum _{|n|\le N} d\beta _{n}(t)e^{in\cdot x}$, (3.5) is then a finite dimensional system of SDEs for the Fourier coefficients $\{ (a_0, a_{n,1},a_{n,2}),(b_0, b_{n,1},b_{n,2})\}_{n\in \Lambda _{N}^{*}}$. We use (4.5) as a bijection between $(u_N,v_N)$ and the coefficients $\{ (a_0, a_{n,1},a_{n,2}),(b_0, b_{n,1},b_{n,2})\}_{n\in \Lambda _{N}^{*}}$. We write (3.5) in the Ito formulation:

5.1 $$\begin{aligned} d \begin{pmatrix}{u_N}\\ {v_N} \end{pmatrix} = \bigg \{ J\nabla _{(u_N, v_N)}H(u_N,v_N) - \begin{pmatrix} {0}\\ {v_N} \end{pmatrix} \bigg \}dt +\sqrt{2} \begin{pmatrix} 0 &  0 \\ 0 &  \Pi _{\le N}\langle \nabla \rangle ^{-s} \end{pmatrix} \begin{pmatrix} {0}\\ {dW_{t}}\end{pmatrix} , \end{aligned}$$

where

$$\begin{aligned} J : = \begin{pmatrix} 0 &  1 \\ -1 &  0 \end{pmatrix}, \end{aligned}$$

and $\nabla _{(u_N,v_N)} = (\partial _{u_N}, \partial _{v_N})$ is the gradient with respect to the variables $(u_N,v_N)$. With respect to $\mathcal {V}_{N} \times \mathcal {V}_N$, we associate to (5.1) the (adjoint of the) infinitesimal generator:

5.2 $$\begin{aligned} \begin{aligned} \mathcal {L}_N^{*} F&= -\text {div}_{(u_N,v_N)} \big [ (J\nabla H)F \big ] +\text {div}_{(u_N,v_N)} \bigg [ \begin{pmatrix} 0 &  0 \\ 0 &  1 \end{pmatrix} \begin{pmatrix} {u_N}\\ {v_N}\end{pmatrix} F \bigg ] \\&+ \text {Tr}\bigg [ D_{(u_N,v_N)}^{2} \begin{pmatrix} 0 &  0 \\ 0 &  \Pi _{\le N}\langle \nabla \rangle ^{-2s} \end{pmatrix}F \bigg ]. \end{aligned} \end{aligned}$$

As $\text {div}_{(u_N,v_N)} J\nabla H=0$, we rewrite (5.2) as

5.3 $$\begin{aligned} \mathcal {L}_N^{*} F&= -\{H, F\} + \text {div}_{v_N}( v_N F) + \text {div}_{v_N}[ \nabla _{v_N}( \langle \nabla \rangle ^{-2s}F)], \end{aligned}$$

where

5.4 $$\begin{aligned} \{H,F\} : = \langle \partial _{v_N}H, \partial _{u_N}F \rangle - \langle \partial _{u_N}H, \partial _{v_N}F \rangle , \end{aligned}$$

and

$$\begin{aligned} \text {div}_{v_N} [ F(v)]: = \text {div}_{(b_0, b_{1,n},b_{2,n})} F(v) = \partial _{b_0} F + \sum _{n=1}^{N} \big ( \partial _{b_{1,n}}F + \partial _{b_{2,n}}F\big ). \end{aligned}$$

Note that

$$\begin{aligned} \text {div}_{v_N} [ v_N F]&= (|\Lambda _{N}^{*}|+1)F + \langle v_N, \partial _{v_N}F \rangle . \end{aligned}$$

Let ${\textbf{u}}_{N}(t)$ denote the almost sure global-in-time solution to (5.1) with initial data ${\textbf{u}}_{0,N}$, as guaranteed by Proposition 3.1, and recall the decompositionThese solutions generate a Markov semigroup

$$\begin{aligned} \widetilde{\mathcal {P}}_{t}F({\textbf{u}}_{0,N}) = \mathbb {E}[ F( {\textbf{u}}_N (t; {\textbf{u}}_0,\xi ) )], \end{aligned}$$

and the push-forward measure $\gamma _{t,N}:=(\widetilde{\mathcal {P}}_{t})_{*} L_N$ is a weak solution to the Fokker-Planck equation:

5.5 $$\begin{aligned} {\left\{ \begin{array}{ll} \partial _t\gamma _{t,N} =\mathcal {L}^{*} \gamma _{t,N} \\ \gamma _{t,N} \vert _{t=0} = e^{-E_{s,N}(u_N,v_N)} dL_{N}, \end{array}\right. } \end{aligned}$$

where $E_{s,N}$ is as in (4.10). See [4, Proposition 1.3.1].

#### Lemma 5.1

For each $N\in 2^{\mathbb {N}_0}$, there exists $h_{t}^{N} \in C^{1,2}([0,\infty )\times \mathbb {R}^{2(|\Lambda _{N}^{*}|+1)})$ such that $\gamma _{t,N} = h_{t}^{N} dL_{N}$, and $h_{t}^{N}$ satisfies

5.6 $$\begin{aligned} \partial _th_{t}^{N}({\textbf{u}}_{0,N}) \vert _{t=0} = \mathcal {L}^{*} ( e^{-E_{s,N}({\textbf{u}}_{0,N})})=-\{H, \mathcal {E}_{s,N}\}({\textbf{u}}_{0,N})e^{-E_{s,N}({\textbf{u}}_{0,N})}, \end{aligned}$$

and

5.7 $$\begin{aligned} \{ H,\mathcal {E}_{s,N}\}({\textbf{u}}_{0,N})= \langle v_N, 3\langle \nabla \rangle ^{s}[ \langle \nabla \rangle ^s u_N \cdot u_N^2]-\langle \nabla \rangle ^{2s}(u_N^3) + 3Q_{s,N}(u_N)u_N \rangle . \end{aligned}$$

#### Proof

As the coefficients of the operator $\mathcal {L}^{*}$ are smooth and the initial datum $e^{-E_{s,N}({\textbf{u}}_{0,N})}$ is smooth, Hörmander’s condition ensures that $\gamma _{t,N}=h_{t}^{N}dL_{N}$ and $h_{t}^{N}\in C^{\infty , \infty }([0,\infty )\times \mathbb {R}^{2(|\Lambda _{N}^{*}|+1)})$. The first equality in (5.6) follows from (5.5) and the second equality follows from (5.3), (5.4), (4.10), and the fact that $\{H,H\}=0$. In particular, we have

$$\begin{aligned} \text {div}_{v_N}[ \nabla _{v_N}( \langle \nabla \rangle ^{-2s}e^{-E_{s,N}})]&=-(|\Lambda _{N}^{*}|+1)e^{-E_{s,N}} - \langle v_N, \partial _{v_N}e^{-E_{s,N}} \rangle \\&=- \text {div}_{v_N}( v_N e^{-E_{s,N}}). \end{aligned}$$

Finally, for (5.7), a direct computation from (4.11) shows that

$$\begin{aligned} \partial _{u_N}\mathcal {E}_{s,N} (u_N,v_N)&= (\langle \nabla \rangle ^{2s}-1)\langle \nabla \rangle ^{2}u_{N}+ 3 \langle \nabla \rangle ^{s}( \langle \nabla \rangle ^{s}u_N \cdot u_N^2) +3 Q_{s,N}(u_N) u_N, \\ \partial _{v_N}\mathcal {E}_{s,N} (u_N,v_N)&= \langle \nabla \rangle ^{2s}v_N-v_{N}, \end{aligned}$$

and from (3.13), we have

$$\begin{aligned} \partial _{u_N}H(u_N,v_N) = u_N - \Delta u_N +u_N^3 \quad \text {and} \quad \partial _{v_N}H(u_N,v_N) = v_N. \end{aligned}$$

Then (5.7) follows from (5.4), and using that $\langle \nabla \rangle ^{2}=1-\Delta$. $\square$

We define

5.8 $$\begin{aligned} g_{t}^{N} ({\textbf{u}}_{0,N}) := e^{E_{s,N}({\textbf{u}}_{0,N})} h_{t}^{N} ({\textbf{u}}_{0,N}), \end{aligned}$$

and we extend $g_{t}^{N}$ to all of $X^{\alpha }$ by defining $g_{t}^{N}({\textbf{u}}_0):= g_{t}^{N}(\Pi _{\le N}{\textbf{u}}_0)$. From Lemma 5.1, we have that $g_{t}^{N}$ is the transported density of the truncated probability measure

5.9 $$\begin{aligned} d\vec {\rho }_{s,N}: = \widetilde{Z}_{s,N}^{-1} e^{-E_{s,N}({\textbf{u}}_{0,N})} dL_{N}= Z_{s,N}^{-1} e^{-R_{s,N}({\textbf{u}}_0)} d\vec {\mu }_{s,N}, \end{aligned}$$

in the sense that for every bounded Borel functional *F*, we have that

5.10 $$\begin{aligned} \int \mathbb {E}[ F(\Phi _{t}^{N}({\textbf{u}}_0,\xi )) ] d\vec {\rho }_{s,N}({\textbf{u}}_0) = \int F({\textbf{u}}_0)g_{t}^{N}({\textbf{u}}_0) d\vec {\rho }_{s,N}({\textbf{u}}_0). \end{aligned}$$

In the following key result, we show that $g_{t}^{N}$ is also the transported density of the measure $(\mathcal {P}_{t}^{N})^{*}\vec {\nu }_{s,N}$ with respect to $\vec {\nu }_{s,N}$, as well as obtain good long-time $L^p$-bounds for $g_{t}^{N}$ when restricted to compact sets.

#### Lemma 5.2

Let $s>0$ and $N\in 2^{\mathbb {N}_0}$. Then, it holds that

5.11 $$\begin{aligned} g_{t}^{N}({\textbf{u}}_0) = \frac{d (\mathcal {P}_{t}^{N})^{*}\vec {\nu }_{s,N} }{ d\vec {\nu }_{s,N}}. \end{aligned}$$

Moreover, for $\alpha >0$ such that $s-\alpha <\frac{1}{3}\min (1,s)$ and $K\subseteq \mathbb {R}^{2(\Lambda _{N}^{*}+1)}$ a compact set, there exists $0<\tau (K,N)\le 1$ such that

5.12 $$\begin{aligned} |g_{t}^{N}({\textbf{u}}_{0,N})|\le 1+ t ( \{H,\mathcal {E}_{s,N}\}({\textbf{u}}_{0,N})+1) \end{aligned}$$

for all $0\le t \le \tau (K,N)$ and ${\textbf{u}}_{0,N}\in K$. Furthermore, given $R>0$, let

5.13 $$\begin{aligned} K_{R}:= \Big \{ {\textbf{u}}_{0}\,:\, \sup _{N\in \mathbb {N}}\big ( \Vert \Pi _{\le N} {\textbf{u}}_{0}\Vert _{\mathcal {C}^{\alpha }}+\Vert Q_{s,N}(\pi _1 {\textbf{u}}_{0})\Vert _{H^{\alpha -s}} \big )\le R\Big \} \end{aligned}$$

and $K_{R,N}:=\Pi _{\le N} K_{R}$. Then, $K_{R,N}$ is pre-compact in $\mathbb {R}^{2(|\Lambda _{N}^{*}|+1)}$ and

5.14 $$\begin{aligned} \sup _{0\le t \le \tau (K_{R,N},N)}\Vert g^{N}_{t}({\textbf{u}}_0) \textbf{1}_{K_{R,N}}(\Pi _{\le N}{\textbf{u}}_0) \Vert _{L^{p}( \vec {\nu }_{s,N})}^{p} \le c_0 e^{ C(R)(p\tau (K_{R,N},N))^2} \end{aligned}$$

for all $1\le p <\infty$, and where the constants $c_0, C(R)$ do not depend on $N\in 2^{\mathbb {N}_0}$.

#### Proof

We first show (5.11). Recalling (4.14) and (4.4), we have

5.15 $$\begin{aligned} d\vec {\nu }_{s,N} =\vec {\rho }_{s,N}\otimes \vec {\mu }_{s,N}^{\perp }. \end{aligned}$$

Let $A \subseteq \Pi _{\le N}X^{\alpha }$ and $B\subseteq \Pi _{>N}X^{\alpha }$ be measurable sets. Then, as $\Pi _{\le N} \xi$ and $\Pi _{>N}\xi$ are independent and, for fixed ${\textbf{u}}_0$, $\Pi _{\le N}\Phi _{t}^{N}({\textbf{u}}_0,\xi )$ and $\Pi _{>N} \Phi ^{\text {lin}}_{t}({\textbf{u}}_0,\xi )$ depend only on $\Pi _{\le N} \xi$ and $\Pi _{>N}\xi$, respectively, the random variables $\Pi _{\le N}\Phi _{t}^{N}({\textbf{u}}_0,\xi )$ and $\Pi _{>N} \Phi ^{\text {lin}}_{t}({\textbf{u}}_0,\xi )$ are independent. Thus, using (5.15) and the invariance of $\vec {\mu }_{s,N}^{\perp }$ under the linear high-frequency flow $\Pi _{>N} \Phi ^{\text {lin}}_{t}({\textbf{u}}_0,\xi )$, as well as (5.10), we obtain

$$\begin{aligned} \int&\textbf{1}_{A}(\Pi _{\le N}{\textbf{u}}_0) \textbf{1}_{B}(\Pi _{>N}{\textbf{u}}_0) d(\mathcal {P}_{t}^{N})^{*}(\vec {\nu }_{s,N}) \\&= \int \mathbb {E}\big [ \textbf{1}_{A}( \Pi _{\le N}\Phi _{t}^{N}({\textbf{u}}_0,\xi )) \textbf{1}_{B}( \Pi _{>N} \Phi _{t}^{\text {lin}}({\textbf{u}}_0,\xi ))\big ] d\vec {\nu }_{s,N} \\&= \int \mathbb {E}[ \textbf{1}_{A}( \Pi _{\le N}\Phi _{t}^{N}({\textbf{u}}_0,\xi )) ] \mathbb {E}[\textbf{1}_{B}( \Pi _{>N} \Phi _{t}^{\text {lin}}({\textbf{u}}_0,\xi ))] d\vec {\nu }_{s,N} \\&= \int \mathbb {E}[ \textbf{1}_{A}( \Pi _{\le N}\Phi _{t}^{N}({\textbf{u}}_0,\xi )) ] \int \mathbb {E}[\textbf{1}_{B}( \Pi _{>N} \Phi _{t}^{\text {lin}}({\textbf{u}}_0,\xi ))] d\vec {\nu }_{s,N}^{\perp } d\vec {\rho }_{s,N} \\&= \int \textbf{1}_{B}(\Pi _{> N}{\textbf{u}}_0) \int \mathbb {E}[ \textbf{1}_{A}( \Pi _{\le N}\Phi _{t}^{N}({\textbf{u}}_0,\xi )) ] d\vec {\rho }_{s,N} d\vec {\mu }_{s,N}^{\perp } \\&= \int \textbf{1}_{A}(\Pi _{\le N}{\textbf{u}}_0) \textbf{1}_{B}(\Pi _{>N}{\textbf{u}}_0) g_{t}^{N}({\textbf{u}}_0) d\vec {\nu }_{s,N}. \end{aligned}$$

As this holds for all such sets *A* and *B*, we conclude (5.11).

We now move onto the latter claims in the lemma. By Lemma 5.1 and (5.8), $g_{t}^{N}\in C^{1,2}([0,\infty )\times \mathbb {R}^{2(|\Lambda _{N}^{*}|+1)})$ and satisfies $g_{0}^{N}({\textbf{u}}_{0,N})=1$ and $\partial _tg_{t}^{N}({\textbf{u}}_{0,N})\vert _{t=0} = -\{H,\mathcal {E}_{s,N}\}({\textbf{u}}_{0,N})$. As $\partial _tg_{t}({\textbf{u}}_{0,N})$ is uniformly continuous on $[0,1]\times K$, there exists $0<\tau (K,N)\le 1$ such that for all $0\le t \le \tau (K,N)$, and ${\textbf{u}}_{0,N}\in K$,

5.16 $$\begin{aligned} |\partial _tg_{t}^{N}({\textbf{u}}_{0,N}) - \partial _tg_{t}^{N}({\textbf{u}}_{0,N})\vert _{t=0}| \le 1. \end{aligned}$$

For ${\textbf{u}}_{0,N}\in K$, and $0\le t\le \tau (K,N)$, Taylor’s theorem implies

$$\begin{aligned} g_{t}^{N}({\textbf{u}}_{0,N})= 1+ t \big [ (\partial _tg_{t}^{N}({\textbf{u}}_{0,N}))\vert _{t=0}+(\partial _tg_{t}^{N}({\textbf{u}}_{0,N}))\vert _{t=t'}-(\partial _tg_{t}^{N}({\textbf{u}}_{0,N}))\vert _{t=0} \big ], \end{aligned}$$

for some $0\le t'\le \tau (K,N)$. Then, (5.12) follows by using (5.16).

We move onto the claims regarding the set $K_{R,N}$. Since the $\mathcal {C}^{\alpha }$ norm controls the Fourier coefficients of ${\textbf{u}}_{0,N}$, $K_{R,N}$ is bounded, and hence pre-compact. We now show the bound (5.14). Let $\tau _{R}:=\tau (K_{R,N},N)$. From (5.12), the inequality $1+x\le e^{x}$ for any $x\in \mathbb {R}$, (4.12), (4.4) and Cauchy-Schwarz, we have

5.17 $$\begin{aligned} \Vert g^{N}_{t}({\textbf{u}}_{0})&\textbf{1}_{K_{R,N}}(\Pi _{\le N}{\textbf{u}}_{0}) \Vert _{L^{p}( \vec {\nu }_{s,N})}^{p}\nonumber \\&\le \int |1+\tau _{R} ( \{H,\mathcal {E}_{s,N}\}({\textbf{u}}_{0,N})+1) |^{p} \textbf{1}_{K_{R,N}}({\textbf{u}}_{0,N}) d\vec {\nu }_{s,N} \nonumber \\&\le \int e^{p \tau _{R}( \{H,\mathcal {E}_{s,N}\}({\textbf{u}}_{0,N})+1)}\textbf{1}_{K_{R,N}}({\textbf{u}}_{0,N}) d\vec {\nu }_{s,N} \nonumber \\&\le Z_{s,N}^{-1} e^{p\tau _{R}} \Vert e^{-R_{s,N}({\textbf{u}})}\Vert _{L^{2}(\vec {\mu }_{s})} \bigg ( \int e^{2 p\tau _{R} \{H,\mathcal {E}_{s,N}\}({\textbf{u}}_{0,N})}\textbf{1}_{K_{R,N}}({\textbf{u}}_{0,N}) d\vec {\mu }_{s,N} \bigg )^{\frac{1}{2}}. \end{aligned}$$

Recall that for $\phi \in C^{\infty }(\mathbb {T}^2)$, the random variable $\langle v_{N}, \phi \rangle$ is a centered complex-valued Gaussian with variance $\Vert \Pi _{\le N}\phi \Vert _{H^{-s}(\mathbb {T}^2)}^{2}$. Moreover from (5.7), $\{H,\mathcal {E}_{s,N}\}$ can be written as $\langle \langle \nabla \rangle ^{s}v_N, \mathcal {W}(u_N) \rangle$, where

$$\begin{aligned} \mathcal {W}(u_N)= 3 [ \langle \nabla \rangle ^s u_N \cdot u_N^2]-\langle \nabla \rangle ^{s}(u_N^3) + 3 \langle \nabla \rangle ^{-s}(Q_{s,N}(u_N)u_N). \end{aligned}$$

Thus, from (5.7) and the independence of the random variables $v_{N}$ and $u_{N}$, we have

$$\begin{aligned}&\int e^{2 p \tau _{R} \{H,\mathcal {E}_{s,N}\}({\textbf{u}}_{0,N})}\textbf{1}_{K_{R,N}}({\textbf{u}}_{0,N}) d\vec {\mu }_{s,N}\\&\le \int \textbf{1}_{\pi _1 K_{R,N}}(u_{N}) \int e^{2 p \tau _{R} \{H,\mathcal {E}_{s,N}\}({\textbf{u}}_{0,N})}d{\mu }_{s,N} (v_N) d\mu _{s+1,N}(u_N) \\&= \int \textbf{1}_{\pi _1 K_{R,N}}(u_{N})e^{ 2(p\tau _{R})^2 \Vert \mathcal {W}(u_N)\Vert _{L^2}^{2} } d\mu _{s+1,N}(u_N). \end{aligned}$$

It follows from (2.12), (2.20), and the definition of $K_{R,N}$, that there exists $C>0$ such that

$$\begin{aligned} \Vert \mathcal {W}(u_N)\Vert _{L^2}^{2} \le C(R^6+R^4). \end{aligned}$$

for $u_{N}\in \pi _1 K_{R,N}$. Inserting this bound back into (5.17) and using (4.17) yields (5.14). $\square$

### Lifted Markov semigroups

For $\varepsilon >0$ and $0<\alpha <s$, we define the spacewith the normIt is clear from (2.38) that $(\mathcal {Z}, \Vert \cdot \Vert _{\mathcal {Z}})$ is a Polish space. We view $\Xi : = \text {Law}( \textbf{1}_{[0,\infty )\times \mathbb {T}^2}\xi )$ as a probability measure on $\mathcal {Z}$. More precisely, for any $F:\mathcal {Z}\rightarrow \mathbb {R}$, we have

$$\begin{aligned} \int F(u)d\Xi (u) =\mathbb {E}[ F(\xi )]. \end{aligned}$$

In the following proposition, we show that for each $N\in 2^{\mathbb {N}_0}\cup \{\infty \}$, the Markov semigroups $\{\mathcal {P}_{t}^{N}\}_{t\ge 0}$ lift to Markov semigroups on the product space $( X^\alpha \times \mathcal {Z}, \vec {\nu }_{s,N}\otimes \Xi )$.

#### Proposition 5.3

Given $N\in 2^{\mathbb {N}_0}\cup \{\infty \}$, there exists a Markov semigroup $\mathcal {U}^{N}_{t}$ on $X^\alpha \times \mathcal {Z}$ such that

5.18 $$\begin{aligned} (\mathcal {P}_{t}^{N})^{*}\vec {\nu }_{s,N} \otimes \Xi = (\mathcal {U}_{t}^{N})^{*}( \nu _{s,N}\otimes \Xi ). \end{aligned}$$

Moreover, the Radon-Nikodym derivative

5.19 $$\begin{aligned} f_{t}^{N}({\textbf{u}}_0, \xi ) : = \frac{d (\mathcal {U}_{t}^{N})^{*}( \vec {\nu }_{s,N}\otimes \Xi )}{d (\vec {\nu }_{s,N}\otimes \Xi )}. \end{aligned}$$

exists and is equal to $g_{t}^{N}({\textbf{u}}_0)$ for almost every $({\textbf{u}}_{0},\xi )$ with respect to $\vec {\nu }_{s,N}\otimes \Xi$, where $g_{t}^{N}$ was defined in (5.8).

#### Proof

For $F\in \mathcal {B}_{b}( X^\alpha \times \mathcal {Z})$, we define the operator $\mathcal {U}^N_{t}:\mathcal {B}_{b}( X^\alpha \times \mathcal {Z})\rightarrow \mathcal {B}_{b}( X^\alpha \times \mathcal {Z})$ by

5.20 $$\begin{aligned} \mathcal {U}_{t}^{N}F ({\textbf{u}}_0,\xi ) = F(\Phi _{t}^{N}({\textbf{u}}_0; \xi ), R_{t}( \textbf{1}_{[t,\infty )}\xi )), \end{aligned}$$

and claim that $\{\mathcal {U}_{t}^{N}\}_{t\ge 0}$ is a Markov semigroup. We only need to check the semigroup property. Note that $R_{t}( \textbf{1}_{[t,\infty )}\xi )= \textbf{1}_{[0,\infty )}R_{t}\xi$ for any $t\in \mathbb {R}$. Given $t_1,t_2\ge 0$, and using (3.17) and (3.18), we have

$$\begin{aligned} \mathcal {U}_{t_1+t_2}^{N}F ({\textbf{u}}_0 ,\xi )&=F( \Phi ^{N}_{t_1+t_2}({\textbf{u}}_0;\xi ), R_{t_1+t_2}(\textbf{1}_{[t_1+t_2,\infty )}\xi )) \\&= F( \Phi ^{N}_{t_1}( \Phi ^{N}_{t_2}({\textbf{u}}_0; \textbf{1}_{[0,t_2]}\xi ); R_{t_2}(\textbf{1}_{[t_2,\infty )}\xi )), R_{t_1} \textbf{1}_{[t_1,\infty )}R_{t_2}\xi ) \\&=F( \Phi ^{N}_{t_1}( \Phi ^{N}_{t_2}({\textbf{u}}_0;\xi ); \textbf{1}_{[0,\infty )}R_{t_2}\xi ), R_{t_1} \textbf{1}_{[t_1,\infty )} \textbf{1}_{[0,\infty )}R_{t_2}\xi )\\&= \mathcal {U}_{t_1}^{N}\big ( F (\Phi ^N_{t_2}({\textbf{u}}_0;\xi ), R_{t_2}\textbf{1}_{[t_2,\infty )}\xi )\big ) \\&= (\mathcal {U}^{N}_{t_1}\circ \mathcal {U}_{t_2}^{N}) F ({\textbf{u}}_0,\xi ). \end{aligned}$$

This proves the semigroup property.

We now show (5.18) and, at the same time, that this density is equal to $g_{t}^{N}$. Note that for any $t >0$, $\textbf{1}_{[0,t]}\xi$ and $R_{t}\textbf{1}_{[t,\infty )}\xi$ are independent. Then, given $A\subseteq X^{\alpha }$ and $B\subseteq \mathcal {Z}$ be measurable and using (5.11), we have

5.21 $$\begin{aligned}&\int \textbf{1}_{A\times B}({\textbf{u}}_0,\xi ) d(\mathcal {U}_{t}^{N})^{*}(\vec {\nu }_{s,N}\otimes \Xi ) \nonumber \\&\quad =\int \textbf{1}_{A}(\Phi _{t}^{N}({\textbf{u}}_0,\xi )) \textbf{1}_{B}(R_{t} \textbf{1}_{[t,\infty )}\xi ) d\Xi d\vec {\nu }_{s,N} \nonumber \\&\quad = \int \mathbb {E}\bigg [ \textbf{1}_{A}(\Phi ^{N}_{t}({\textbf{u}}_0, \textbf{1}_{[0,t]}\xi )) \textbf{1}_{B}( R_{t} \textbf{1}_{[t,\infty )}\xi ) \bigg ] d\vec {\nu }_{s,N} \nonumber \\&\quad = \int \mathbb {E}\Big [ \textbf{1}_{A}(\Phi ^{N}_{t}({\textbf{u}}_0, \textbf{1}_{[0,t]}\xi )) \Big ] \mathbb {E}\Big [ \textbf{1}_{B}( R_{t} \textbf{1}_{[t,\infty )}\xi ) \Big ] d\vec {\nu }_{s,N} \nonumber \\&\quad = \int \textbf{1}_{A\times B}({\textbf{u}}_0, \xi ) d((\mathcal {P}_{t}^{N})^{*}\vec {\nu }_{s,N}\otimes \Xi ) \nonumber \\&\quad = \int \textbf{1}_{A\times B}({\textbf{u}}_0, \xi ) g_{t}^{N}({\textbf{u}}_0) d(\vec {\nu }_{s,N}\otimes \Xi ). \end{aligned}$$

As this identity is true for all such measurable sets *A* and *B*, we get that $f_{t}^{N}= g_{t}^{N}$ a.e. $\square$

In order to establish the absolute continuity result, we need to argue locally in the function spaces, which necessitates keeping track of the flow. For a fixed measurable set $K\subseteq X^{\alpha }$ and $t\ge 0$, we define

5.22 $$\begin{aligned} E_{t}^{N,K} : = \big \{ ({\textbf{u}}_0, \xi ) \,:\, \Phi ^{N}_{t'}({\textbf{u}}_0,\xi )\in K \quad \text {for all} \,\, 0\le t'\le t\big \} \end{aligned}$$

These sets behave well with respect to the Markov semigroup $\mathcal {U}_{t}^{N}$ in the following sense.

#### Lemma 5.4

For every $t,\tau \ge 0$, and $K\subseteq X^\alpha$ measurable, it holds that

5.23 $$\begin{aligned} \textbf{1}_{E_{t+\tau }^{N,K}}({\textbf{u}}_0,\xi ) = \mathcal {U}^{N}_{\tau }( \textbf{1}_{E_{t}^{N,K}})({\textbf{u}}_0 ,\xi )\textbf{1}_{E_{\tau }^{N,K}}({\textbf{u}}_0,\xi ). \end{aligned}$$

#### Proof

Suppose that $({\textbf{u}}_0, \xi ) \in E_{t+\tau }^{N,K}$. Using (5.20), (5.22), and (3.18), we have

$$\begin{aligned}&\mathcal {U}^{N}_{\tau }( \textbf{1}_{E_{t}^{N,K}})({\textbf{u}}_0 ,\xi ) = \textbf{1}_{E_{t}^{N,K}}( \Phi _{\tau }^{N}({\textbf{u}}_0,\xi ), R_{\tau }(\textbf{1}_{[\tau ,\infty )}\xi ))\\&\quad = \textbf{1}_{\{ \Phi _{t'}^{N}({\textbf{u}}_0,\xi )\in K \,\, \forall \,\,t'\in [\tau ,\tau +t]\}}({\textbf{u}}_0, \xi )=1. \end{aligned}$$

Moreover, by the inclusion

5.24 $$\begin{aligned} E_{t_1}^{N,K}\subseteq E_{t_2}^{N,K} \end{aligned}$$

for any $0\le t_2\le t_1$, we have that $\textbf{1}_{E_{\tau }^{N,K}}({\textbf{u}}_0,\xi ) =1$.

Next, assume that $({\textbf{u}}_0,\xi )\notin E_{t+\tau }^{N,K}$. Then, there is a $t_0 \in [0,\tau +t]$ such that $\Phi _{t_0}^{N}({\textbf{u}}_0, \xi )\notin K$. If $t_0 \in [0,\tau ]$, then $\textbf{1}_{E_{\tau }^{N,K}}({\textbf{u}}_0,\xi )=0$. If instead $t_0\in (\tau ,\tau +t]$, then $\mathcal {U}^{N}_{\tau }( \textbf{1}_{E_{t}^{N,K}})({\textbf{u}}_0,\xi )=0$. $\square$

We now obtain an analogue of Proposition 5.3 for the localised measures $(\vec {\nu }_{s,N}\otimes \Xi ) \textbf{1}_{E_{t}^{N,K}}$.

#### Lemma 5.5

Let $N\in \mathbb {N}$, $K\subseteq X^\alpha$ be measurable, $t\ge 0$, and $E_t^{N,K}$ be as in (5.22). Then, the measure $(\mathcal {U}_{t}^{N})^{*}( (\vec {\nu }_{s,N}\otimes \Xi ) \textbf{1}_{E_{t}^{N,K}})$ has a density with respect to the probability measure $\vec {\nu }_{s,N}\otimes \Xi$ and we set

5.25 $$\begin{aligned} f_{t}^{N,K}({\textbf{u}}_0, \xi ) : = \frac{d (\mathcal {U}_{t}^{N})^{*}( (\vec {\nu }_{s,N}\otimes \Xi ) \textbf{1}_{E_{t}^{N,K}})}{d (\vec {\nu }_{s,N}\otimes \Xi )}. \end{aligned}$$

In particular, the following properties hold:

(i) $f_{t}^{N,K}({\textbf{u}}_0,\xi ) \ge 0$ for $\vec {\nu }_{s,N}\otimes \Xi$ almost every $({\textbf{u}}_0,\xi )$,

(ii) $f_{t}^{N,K}({\textbf{u}}_0,\xi ) = 0$ for almost every $({\textbf{u}}_0,\xi )\in (X^{\alpha }\setminus K)\times \mathcal {Z}$,

(iii) $f_{t}^{N,K}({\textbf{u}}_0,\xi ) \le g_{t}^{N} ({\textbf{u}}_0)$ for almost every $({\textbf{u}}_0,\xi )$.

#### Proof

The existence of (5.25) and consequently (i) is immediate from the existence of $f_{t}^{N}$ which we showed in Proposition 5.3. We move onto showing the properties (ii) and (iii). First, by the definition (5.25) we have

5.26 $$\begin{aligned} \int F({\textbf{u}}_0,\xi ) f_{t}^{N,K}({\textbf{u}}_0,\xi ) d(\vec {\nu }_{s,N}\otimes \Xi )&= \int (\mathcal {U}^{N}_{t}F)({\textbf{u}}_0, \xi ) \textbf{1}_{E_{t}^{N,K}}({\textbf{u}}_0,\xi ) d(\vec {\nu }_{s,N}\otimes \Xi ) \end{aligned}$$

for any $F\in \mathcal {B}_{b}(X^{\alpha }\times \mathcal {Z})$. By choosing $F=\textbf{1}_{X^{\alpha }\setminus K}({\textbf{u}}_0)$ in (5.26) and noting that $E_{t}^{N,K}$ is disjoint from $\{\Phi ^{N}_{t}({\textbf{u}}_0,\xi )\in (X^{\alpha }\setminus K)\times \mathcal {Z}\}$, we obtain

5.27 $$\begin{aligned} \int \textbf{1}_{X^{\alpha }\setminus K}({\textbf{u}}_0) f_{t}^{N,K}({\textbf{u}}_0,\xi ) d(\vec {\nu }_{s,N}\otimes \Xi )=0. \end{aligned}$$

Property (ii) now follows from property (i) and that $f_{t}^{N,K}\ge 0$.

We move onto showing property (iii). For $F\in \mathcal {B}_{b}(X^{\alpha }\times \mathcal {Z})$ such that $F\ge 0$, (5.26), (5.24) and (5.20), we have

5.28 $$\begin{aligned} \begin{aligned} \int F({\textbf{u}}_0,\xi ) f_{t}^{N,K}({\textbf{u}}_0,\xi ) d(\vec {\nu }_{s,N}\otimes \Xi )&= \int (\mathcal {U}^{N}_{t}F)({\textbf{u}}_0, \xi ) \textbf{1}_{E_{t}^{N,K}}({\textbf{u}}_0,\xi ) d(\vec {\nu }_{s,N}\otimes \Xi ) \\&\le \int (\mathcal {U}^{N}_{t}F)({\textbf{u}}_0, \xi ) \textbf{1}_{K}(\Phi _{t}^{N}({\textbf{u}}_0,\xi )) d(\vec {\nu }_{s,N}\otimes \Xi ) \\&= \int \mathcal {U}^{N}_{t}(F \textbf{1}_{K})({\textbf{u}}_0, \xi ) d(\vec {\nu }_{s,N}\otimes \Xi ) \\&= \int F({\textbf{u}}_0,\xi ) \textbf{1}_{K}({\textbf{u}}_0) f_{t}^{N}({\textbf{u}}_0,\xi )d(\vec {\nu }_{s,N}\otimes \Xi ) \\&= \int F({\textbf{u}}_0,\xi ) \textbf{1}_{K}({\textbf{u}}_0) g_{t}^{N}({\textbf{u}}_0)d(\vec {\nu }_{s,N}\otimes \Xi ), \end{aligned} \end{aligned}$$

which proves (iii). $\square$

### $L^p$ bounds on densities

The key ingredient for the proof of Theorem 1.1 is the following long-time $L^p$ bounds on the truncated densities $f_{t}^{N,K}$.

#### Proposition 5.6

(Uniform long-time bounds on densities) Let $s>0$, $0<\alpha <s$ be as in Lemma 5.2, $T>0$, $R>0$, and $K_{R}$ be as in (5.13). Then, it holds that

5.29 $$\begin{aligned} \sup _{N\in 2^{\mathbb {N}_0}} \sup _{t\in [0,T]} \Vert f_{t}^{N,K_{R}} \Vert _{L^{q} (\vec {\nu }_{s,N}\otimes \Xi )} \le C(R,T,q) \end{aligned}$$

for any $$1\le q<\infty $$.

#### Proof

To simplify the notation, we write *K* in place of $K_{R}$. First, we will prove that

5.30 $$\begin{aligned} \begin{aligned} \Vert f_{t+\tau }^{N,K}\Vert _{L^{r}(\vec {\nu }_{s,N}\otimes \Xi )} \le \big \Vert f_{\tau }^{N,K} \big \Vert _{L^p (\vec {\nu }_{s,N}\otimes \Xi )} \big \Vert f_{t}^{N,K}\big \Vert _{L^{\frac{r(p-1)}{p-r}}(\vec {\nu }_{s,N}\otimes \Xi )}^{\frac{1}{p'}}, \end{aligned} \end{aligned}$$

for any $0<\tau ,t<T$ such that $t+\tau \le T$ and $1\le r<p<\infty$.

Let $F\in C_{b}( X^\alpha \times \mathcal {Z})$. Then, by (5.23), the semigroup property of $\mathcal {U}_{t}^{N}$, (5.24), and Hölder’s inequality,

5.31 $$\begin{aligned} \int&F({\textbf{u}}_0 , \xi ) f_{t+\tau }^{N,K}({\textbf{u}}_0,\xi )d( \vec {\nu }_{s,N}\otimes \Xi ) \nonumber \\&= \int (\mathcal {U}^N_{t+\tau }F )({\textbf{u}}_0, \xi ) \textbf{1}_{E^{N,K}_{t+\tau }}({\textbf{u}}_0, \xi ) d ( \vec {\nu }_{s,N}\otimes \Xi ) \nonumber \\&= \int \mathcal {U}^{N}_{\tau }( \mathcal {U}^{N}_{t}F)({\textbf{u}}_0,\xi )\mathcal {U}^{N}_{\tau }(\textbf{1}_{E^{N,K}_{t}})({\textbf{u}}_0,\xi ) \textbf{1}_{E^{N,K}_{\tau }}({\textbf{u}}_0,\xi ) d( \vec {\nu }_{s,N}\otimes \Xi ) \nonumber \\&= \int \mathcal {U}^{N}_{\tau }(( \mathcal {U}^{N}_{t}F)\textbf{1}_{E^{N,K}_{t}})({\textbf{u}}_0,\xi ) \textbf{1}_{E^{N,K}_{\tau }}({\textbf{u}}_0,\xi ) d( \vec {\nu }_{s,N}\otimes \Xi ) \nonumber \\&= \int f_{\tau }^{N,K}({\textbf{u}}_0,\xi )(\mathcal {U}^{N}_{t}F)({\textbf{u}}_0,\xi )\textbf{1}_{E^{N,K}_{t}}({\textbf{u}}_0,\xi )d ( \vec {\nu }_{s,N}\otimes \Xi ) \nonumber \\&\le \big \Vert f_{\tau }^{N,K}\big \Vert _{L^p ( \vec {\nu }_{s,N}\otimes \Xi ) } \big \Vert (\mathcal {U}^{N}_{t}F)\textbf{1}_{E^{N,K}_{t}}\big \Vert _{L^{p'} ( \vec {\nu }_{s,N}\otimes \Xi ) }. \end{aligned}$$

Now using the definition (5.20) and (5.23), we have

5.32 $$\begin{aligned} \big \Vert (\mathcal {U}^{N}_{t}F )\textbf{1}_{E^{N,K}_{t}}\big \Vert _{L^{q}(\vec {\nu }_{s,N}\otimes \Xi )}^{q}&= \int \mathcal {U}_{t}^{N}(|F|^{q})\textbf{1}_{E_{t}^{N,K}}({\textbf{u}}_0,\xi ) d(\vec {\nu }_{s,N}\otimes \Xi ) \nonumber \\&= \int |F({\textbf{u}}_0,\xi )|^{q} f_{t}^{N,K}({\textbf{u}}_0,\xi )d(\vec {\nu }_{s,N}\otimes \Xi ). \end{aligned}$$

for any $1\le q<\infty$. Using (5.32) in (5.31), we then have

$$\begin{aligned} \int&F({\textbf{u}}_0 , \xi ) f_{t+\tau }^{N,K}({\textbf{u}}_0,\xi )d( \vec {\nu }_{s,N}\otimes \Xi )\\&\le \big \Vert f_{\tau }^{N,K}\big \Vert _{L^p ( \vec {\nu }_{s,N}\otimes \Xi ) } \big \Vert F\cdot (f_{t}^{N,K})^{\frac{1}{p'}}\big \Vert _{L^{p'}( \vec {\nu }_{s,N}\otimes \Xi )}\\&\le \big \Vert f_{\tau }^{N,K}\big \Vert _{L^p ( \vec {\nu }_{s,N}\otimes \Xi ) } \Vert F\Vert _{L^{r'}(\vec {\nu }_{s,N}\otimes \Xi )} \big \Vert (f_{t}^{N,K})^{\frac{1}{p'}}\big \Vert _{L^{\frac{rp}{p-r}} (\vec {\nu }_{s,N}\otimes \Xi )} \\&= \big \Vert f_{\tau }^{N,K}\big \Vert _{L^p ( \vec {\nu }_{s,N}\otimes \Xi ) } \Vert F\Vert _{L^{r'}(\vec {\nu }_{s,N}\otimes \Xi )} \big \Vert f_{t}^{N,K} \big \Vert _{L^{\frac{r(p-1)}{p-r}}(\vec {\nu }_{s,N}\otimes \Xi )}^{\frac{1}{p'}}. \end{aligned}$$

Thus, by duality we establish (5.30).

We now iterate (5.30). We choose the time-step $0<\tau =\tau _{R}:=\tau (K_{R},N)\le 1$ from Lemma 5.2 and break the time interval [0, *T*] into $J=\lceil \frac{T}{\tau } \rceil$ many sub-intervals of width $\tau _{R}>0$. We let $t_{j}:= j\tau _{R}$ for $j=0,1,\ldots , J$, and note that $0\le j\tau _{R} \le T$. For $\varepsilon _0>0$ to be chosen sufficiently small, we define $p:= \frac{1}{\varepsilon _0 \tau _{R}}>1$ and the sequence $\{r_{j}\}_{j=1}^{J}$ recursively by

$$\begin{aligned} r_{1}=3q \quad \text {and} \quad r_{j+1} = \frac{r_{j}\, p}{p+r_{j}-1}. \end{aligned}$$

Note that $1<r_{j+1}<r_{j}$ for all $j=1,\ldots , J-1$ and $r_{j}=\frac{r_{j+1}(p-1)}{p-r_{j+1}}$. Using that the sequence $\{r_{j}\}_{j=1}^{J}$ is decreasing, we have

$$\begin{aligned} r_{1}-r_{J} = \sum _{j=1}^{J-1} (r_{j}-r_{j+1}) =\sum _{j=1}^{J-1} \frac{r_j (r_{j}-1)}{p+r_{j}-1} \le \frac{r_{1}^{2}}{p}J \le r_{1}^{2} \varepsilon _0 T. \end{aligned}$$

Thus, by choosing

$$\begin{aligned} \varepsilon _0=\varepsilon _0(q,T) =\min \big ( \tfrac{1}{2q-1}, \tfrac{\min (9T,1)}{9T q}\big )>0, \end{aligned}$$

we ensure that $p\ge 2q-1\ge q$ and $r_{1}-r_{J}\le q$. In particular, $r_{J}\ge 2q$. For $j=1,\ldots ,J-1$, (5.30) implies

$$\begin{aligned} \Vert f^{N,K}_{t_{j+1}}\Vert _{L^{r_{j+1}}(\vec {\nu }_{s,N}\otimes \Xi )}&\le \Vert f_{\tau _{R}}^{N,K} \Vert _{L^p (\vec {\nu }_{s,N}\otimes \Xi )} \big \Vert f_{t_j}^{N,K}\big \Vert _{L^{r_{j}}(\vec {\nu }_{s,N}\otimes \Xi )}^{\frac{1}{p'}} \end{aligned}$$

Therefore,

$$\begin{aligned} \Vert f^{N,K}_{t_{j}}\Vert _{L^{r_{j}}(\vec {\nu }_{s,N}\otimes \Xi )} \le \Vert f_{\tau _{R}}^{N,K} \Vert _{L^p (\vec {\nu }_{s,N}\otimes \Xi )}^{\sum _{k=0}^{j} \frac{1}{(p')^{k}}}, \end{aligned}$$

for each $j=1,\ldots , J$. As

$$\begin{aligned} \sum _{k=0}^{\infty } \frac{1}{(p')^{k}} = \frac{1}{1-\frac{1}{p}}=p, \end{aligned}$$

Hölder’s inequality then implies

5.33 $$\begin{aligned} \max _{j=1,\ldots , J} \Vert f^{N,K}_{t_{j}}\Vert _{L^{2q}(\vec {\nu }_{s,N}\otimes \Xi )} \le \max \big (1, \Vert f_{\tau _{R}}^{N,K} \Vert _{L^p (\vec {\nu }_{s,N}\otimes \Xi )}^{J}\big ). \end{aligned}$$

Now recalling that we chose *p* such that $p\tau _{R} =\varepsilon _0^{-1}$ and using Lemma 5.5 (ii)-(iii), Proposition 5.3, and (5.14), we have

5.34 $$\begin{aligned} \begin{aligned} \Vert f_{\tau _{R}}^{N,K_{R}} \Vert _{L^p (\vec {\nu }_{s,N}\otimes \Xi )}^{J}&\le \Vert g_{\tau _{R}}^{N} \textbf{1}_{K_{R}}({\textbf{u}}_0) \Vert _{L^{p}(\vec {\nu }_{s,N})}^{J} \\&\le \Vert g_{\tau _{R}}^{N} \textbf{1}_{\Pi _{\le N}K_{R}}(\Pi _{\le N}{\textbf{u}}_0) \Vert _{L^{p}(\vec {\nu }_{s,N})}^{J}\\&= \big ( \Vert g_{\tau _{R}}^{N} \textbf{1}_{K_{R,N}}( \Pi _{\le N}{\textbf{u}}_0) \Vert _{L^{p}(\vec {\nu }_{s,N})}^{p} \big )^{\frac{J}{p}} \\&\le c_{0}^{\varepsilon _0 T} \exp ( C(R) T\varepsilon _0^{-1}) =: \widetilde{C}(R,T,q), \end{aligned} \end{aligned}$$

uniformly in $N\in 2^{\mathbb {N}_0}$. Returning this bound to (5.33), establishes that

5.35 $$\begin{aligned} \max _{j=1,\ldots , J} \Vert f^{N,K}_{t_{j}}\Vert _{L^{2q}(\vec {\nu }_{s,N}\otimes \Xi )} \le \widetilde{C}(R,T,q) \end{aligned}$$

uniformly in $$N\in 2^{\mathbb {N}_0}$$.

Now we obtain $L^q$ bounds for *every* $t\in [0,T]$. Let $t\in [0,T]$ and assume that *t* is not an integer multiple of $\tau _{R}$. If $t\in (0,\tau _{R})$, then Lemma 5.5 (ii)-(iii), Proposition 5.3, (5.14) imply

5.36 $$\begin{aligned} \sup _{t\in [0,\tau _{R}]} \Vert f_{t}^{N,K}\Vert _{L^p (\vec {\nu }_{s,N}\otimes \Xi )} \le \widetilde{C}(R,T,q) \end{aligned}$$

uniformly in $N\in 2^{\mathbb {N}_0}$. Now we assume that $t>\tau _{R}$. There is a $k\in \mathbb {N}$ (depending on *N*) such that $k\tau _{R}<t<(k+1)\tau _{R}$. By repeating the arguments leading to (5.30), we find that

$$\begin{aligned} \Vert f_{t}^{N,K}\Vert _{L^{r}(\vec {\nu }_{s,N}\otimes \Xi )} \le \Vert f_{t-k\tau _{R}}^{N,K}\Vert _{L^p (\vec {\nu }_{s,N}\otimes \Xi )} \Vert f_{k\tau _{R}}^{N,K}\Vert _{L^{{\frac{r(p-1)}{p-r}}}(\vec {\nu }_{s,N}\otimes \Xi )}^{\frac{1}{p'}} \end{aligned}$$

for any $1\le r<p<\infty$. We choose $r=\frac{2qp}{p+2q-1}$ so that $\frac{r(p-1)}{p-r}=2q$. Moreover, since $p\ge 2q-1$, we have $r\ge q$. It now follows from (5.35), (5.36), and Hölder’s inequality that

5.37 $$\begin{aligned} \sup _{N\in 2^{\mathbb {N}_0}}\Vert f_{t}^{N,K}\Vert _{L^{q}(\vec {\nu }_{s,N}\otimes \Xi )} \le \widetilde{C}(R,T,q)^{2} \end{aligned}$$

As $t\in [0,T]$ was arbitrary, (5.36) and (5.37) complete the proof of (5.29). $\square$

### Proof of Theorem 1.1

Let $s>0$, $0<\alpha <s$ be as in Lemma 5.2. Let $T>0$. For $L\ge 1$, define the setand let $0<\alpha '<\alpha$ be sufficiently close to $\alpha$ so that $(s,\alpha ')$ also satisfy the conditions in Lemmas 4.5, 4.6, and 5.2. Then, for any $F\in C_{b}(\mathcal {H}^{\alpha '}(\mathbb {T}^2))$ such that $F\ge 0$, Lemma 3.2, and Lemma 4.1 imply

5.38 $$\begin{aligned} \begin{aligned} \int F({\textbf{u}}_0) d (\mathcal {P}_{t})^{*} \vec {\nu }_s&= \int (\mathcal {P}_{t}F)({\textbf{u}}_0) d\vec {\nu }_{s} \\&=\int \mathbb {E}\big [ F(\Phi _{t}({\textbf{u}}_0,\xi )) \big ] d\vec {\nu }_{s} \\&=\lim _{N\rightarrow \infty } \int \mathbb {E}\big [ F(\Phi ^{N}_{t}({\textbf{u}}_0,\xi )) \big ] d\vec {\nu }_{s} \\&=\lim _{N\rightarrow \infty } \int \mathbb {E}\big [ F(\Phi ^{N}_{t}({\textbf{u}}_0,\xi )) \big ] d\vec {\nu }_{s,N} \\&= \lim _{N\rightarrow \infty } \int F(\Phi _{t}^{N}({\textbf{u}}_0,\xi )) d(\vec {\nu }_{s,N}\otimes \Xi ) \\&\le \limsup _{N\rightarrow \infty } \int \textbf{1}_{D_{T,L}}({\textbf{u}}_0, \xi ) F(\Phi _{t}^{N}({\textbf{u}}_0,\xi )) d(\vec {\nu }_{s,N}\otimes \Xi ) \\&+\limsup _{N\rightarrow \infty } \int \textbf{1}_{D_{T,L}^{c}}({\textbf{u}}_0,\xi ) F(\Phi _{t}^{N}({\textbf{u}}_0,\xi )) d(\vec {\nu }_{s,N}\otimes \Xi ). \end{aligned} \end{aligned}$$

It follows by Markov’s inequality with (3.7), (2.40), (4.20), (4.26) that

5.39 $$\begin{aligned} \bigg |\int \textbf{1}_{D_{T,L}^{c}}({\textbf{u}}_0,\xi ) F(\Phi _{t}^{N}({\textbf{u}}_0,\xi )) d(\vec {\nu }_{s,N}\otimes \Xi ) \bigg | \le \frac{C(T,s,\alpha ) \Vert F\Vert _{L^{\infty }}}{L}. \end{aligned}$$

From Lemma 4.5 and Lemma 4.6, we see that if $({\textbf{u}}_0,\xi )\in D_{T,L}$, then $({\textbf{u}}_0,\xi ) \in E^{N,\widetilde{K}_{C(L,T)}}_{t}$, where $\widetilde{K}_{C(L,T)}$ is a set of the same form as $K_{R}$ in (5.13) but with radius *C*(*L*, *T*) and $\alpha$ replaced by $\alpha '$. Consequently,

5.40 $$\begin{aligned} \begin{aligned} \int&\textbf{1}_{D_{T,L}}({\textbf{u}}_0, \xi ) F(\Phi _{t}^{N}({\textbf{u}}_0,\xi )) d(\vec {\nu }_{s,N}\otimes \Xi ) \\&\le \int \textbf{1}_{E^{N,\widetilde{K}_{C(L,T)}}_{t}}({\textbf{u}}_0,\xi ) F(\Phi _{t}^{N}({\textbf{u}}_0,\xi )) d(\vec {\nu }_{s,N}\otimes \Xi )\\&= \int F({\textbf{u}}_0) f_{t}^{N,\widetilde{K}_{C(L,T)}}({\textbf{u}}_0,\xi ) d(\vec {\nu }_{s,N}\otimes \Xi ). \end{aligned} \end{aligned}$$

It follows from (5.29) that, up to a subsequence,

$$\begin{aligned} f_{t}^{N,\widetilde{K}_{C(R,L,T)}}({\textbf{u}}_0,\xi ) Z_{s,N}^{-1}e^{-R_{s,N}({\textbf{u}}_0)} \end{aligned}$$

converges weakly in $L^2(d(\vec {\mu }_{s}\otimes \Xi ))$ to some element $\mathfrak {f}_{t}^{\widetilde{K}_{C(L,T)}}\in L^2(d(\vec {\mu }_{s}\otimes \Xi ))$. It then follows from (5.38), (5.39), and (5.40) that

5.41 $$\begin{aligned} \int F({\textbf{u}}_0) d (\mathcal {P}_{t})_{*}\vec {\nu }_s \le \int F({\textbf{u}}_0) \mathfrak {f}_{t}^{\widetilde{K}_{C(L,T)}}({\textbf{u}}_0,\xi ) d(\vec {\mu }_{s}\otimes \Xi ) + \tfrac{C(T,s,\alpha )\Vert F\Vert _{L^{\infty }}}{L}. \end{aligned}$$

By density of continuous, bounded functions in

$$\begin{aligned} L^1\big ( (\mathcal {P}_{t})_{*}\vec {\nu }_s+(\pi _{1})_{*} \mathfrak {f}_{t}^{\widetilde{K}_{C(L,T)}}({\textbf{u}}_0,\xi ) d(\vec {\mu }_{s}\otimes \Xi )\big ), \end{aligned}$$

we can extend (5.41) to hold for all $F\in \mathcal {B}_{b}(X^{\alpha })$. Let $E\subset X^{\alpha }$ be a Borel set such that $\vec {\mu }_{s}(E)=0$. Therefore, by (5.41), we obtain

$$\begin{aligned} \int \textbf{1}_{E}({\textbf{u}}_0) d (\mathcal {P}_{t})_{*}\vec {\nu }_s \le \tfrac{C(T,s,\alpha )}{L}, \end{aligned}$$

for every $L\ge 1$. By taking $L\rightarrow \infty$, we obtain that $(\mathcal {P}_{t})_{*}\vec {\nu }_{s}(E)=0$. Since this holds for every *E* with $\vec {\mu }_s(E) = 0$, we deduce that $(\mathcal {P}_{t})_{*}\vec {\mu }_{s} \ll (\mathcal {P}_{t})_{*}\vec {\nu }_{s} \ll \vec {\mu }_s$. This completes the proof of Theorem 1.1.

#### Proof of Theorem 1.2

We write

$$\begin{aligned} \vec {\rho }_s = {\vec {\rho }_s}^{(1)} + {\vec {\rho }_s}^{(2)}, \end{aligned}$$

with ${\vec {\rho }_s}^{(1)} \ll \vec {\mu }_s$ and ${\vec {\rho }_s}^{(1)} \perp \vec {\mu }_s$. The result follows if we show that either ${\vec {\rho }_s}^{(1)} = 0$, or ${\vec {\rho }_s}^{(2)} = 0$. Let *E* be a set such that $\vec {\mu }_s(E) = 1$ and ${\vec {\rho }_s}^{(2)}(E) = 0$. Then, by Theorem 1.1, we have that $\mathcal {P}_{t}(\textbf{1}_E)({\textbf{u}})= \textbf{1}_E({\textbf{u}})$ for $\vec {\mu }_s$-a.e. ${\textbf{u}}$. In particular, recalling that $\textbf{1}_E({\textbf{u}}) = 0$ for ${\vec {\rho }_s}^{(2)}$-a.e. ${\textbf{u}}$, we have that

$$\begin{aligned} \mathcal {P}_{t}(\textbf{1}_E)({\textbf{u}})\ge \textbf{1}_E({\textbf{u}}) \text { for }\vec {\rho }_s\text {-a.e. }{\textbf{u}}. \end{aligned}$$

Since $\int \mathcal {P}_{t}(\textbf{1}_E)({\textbf{u}})d\vec {\rho }_s(u) = \int \textbf{1}_E({\textbf{u}})d\vec {\rho }_s(u)$, we deduce

$$\begin{aligned} \mathcal {P}_{t}(\textbf{1}_E)({\textbf{u}})= \textbf{1}_E({\textbf{u}}) \text { for }\vec {\rho }_s\text {-a.e. }{\textbf{u}}. \end{aligned}$$

In particular,

$$\begin{aligned} (\mathcal {P}_{t})_*({\vec {\rho }_s}^{(1)}) = (\mathcal {P}_{t})_*(\textbf{1}_E {\vec {\rho }_s}) = \textbf{1}_E {\vec {\rho }_s} = {\vec {\rho }_s}^{(1)}. \end{aligned}$$

Since $\vec {\rho }_s$ is the only invariant measure for (1.1), for $\lambda = \Vert {\vec {\rho }_s}^{(1)}\Vert _\mathrm{{TV}}$, we have that ${\vec {\rho }_s}^{(1)} = \lambda \vec {\rho }_s$, and so ${\vec {\rho }_s}^{(2)} = (1-\lambda ) \vec {\rho }_s$ Since ${\vec {\rho }_s}^{(1)} \perp {\vec {\rho }_s}^{(2)}$, this immediately implies $\lambda = 0$ or $\lambda =1$, which concludes the proof. $\square$

## Data Availability

No datasets were generated or analysed during the current study.

## Footnotes

1 Before the recent result, the state-of-the art was that one can show that the invariant measure is absolutely continuous with respect to the stationary law of the linear solution when $\Delta$ is replaced with $-(-\Delta )^{1+\varepsilon }$ for some $\varepsilon > 0$, see [33].

2 The expression above must be considered formally, as $\sigma =+\infty$. Nevertheless, one can define the measure $Z^{-1} \exp (- E(u,v)) dudv$ via a renormalisation procedure.
